# Supplementary material for: Attention-enhanced variational learning for physically informed discovery of exceptionally hard multicomponent bulk metallic glasses
Source: Nat Commun. 2026 May 12;17:4266. doi: 10.1038/s41467-026-73008-0 (PMC13168334; doi:10.1038/s41467-026-73008-0)
Supplement: Supplementary file 1 — Supplementary Information [file 41467_2026_73008_MOESM1_ESM.pdf]

Supplementary Information

Attention-enhanced variational learning for physically informed discovery of exceptionally hard multicomponent bulk metallic glasses

Anurag Bajpai*, Jaemin Wang, Barak Ratzker, Bilgehan Murat Şeșen, Florian Kark, Dierk Raabe*

Max Planck Institute for Sustainable Materials, 40237 Düsseldorf, Germany

***Corresponding Authors:**
Anurag Bajpai (a.bajpai@mpi-susmat.de)
Dierk Raabe (d.raabe@mpi-susmat.de)

This PDF file contains:

1. Supplementary Notes: 1 to 12
2. Supplementary Figures: 1 to 38
3. Supplementary Tables: 1 to 9
4. Supplementary references

Supplementary Note 1. Input Dataset Analysis

We curated a dataset comprising 673 multicomponent metallic glass (MMG) alloy compositions, their associated Vickers hardness (HV) values, and corresponding indentation loads (in Newtons), collected from peer-reviewed literature sources.[1-43] The dataset spans a broad compositional space incorporating transition metals, refractory elements, and rare-earth additions, offering a comprehensive foundation for data-driven hardness prediction and inverse design.

Elemental distribution across the dataset is shown in Supplementary Figure 1, where elements such as B, Fe, Nb, and Zr dominate, consistent with their frequent use in MMG systems due to their known effects on GFA and mechanical performance. Zr-based alloys are widely studied for their high GFA and fracture resistance, while Fe- and Nb-rich systems offer enhanced hardness and thermal stability. The comparatively lower frequencies of rare-earth elements (e.g., Dy, Er, Y) and high-cost elements (e.g., Re, Ru, Pt) reflect their selective usage to modulate cluster chemistry, diffusion kinetics, and phase stability.

The distribution of hardness values across the dataset, presented in Supplementary Figure 2a, shows a multimodal character with peaks around 500 HV and 1200 HV. These modes likely reflect distinct subfamilies of MMGs, for instance, softer Al- and Cu-based glasses and harder Fe- or refractory-rich systems. The widespread occurrence of HV values (ranging from <200 HV to >2200 HV) underscores the structural and topological heterogeneity within MMGs, making hardness prediction a challenging, nonlinear task.

Supplementary Figure 2b plots HV as a function of indentation load. Notably, substantial scatter is observed even under fixed-load conditions, highlighting the impact of load-dependent deformation mechanisms, including the indentation size effect (ISE), pile-up/sink-in behavior, and surface roughness. Furthermore, variability may arise from compositional gradients or differences in short-range ordering across alloy families. This underscores the necessity of treating load as a critical input feature in the machine learning model to enable accurate predictions and generalization across diverse test conditions.

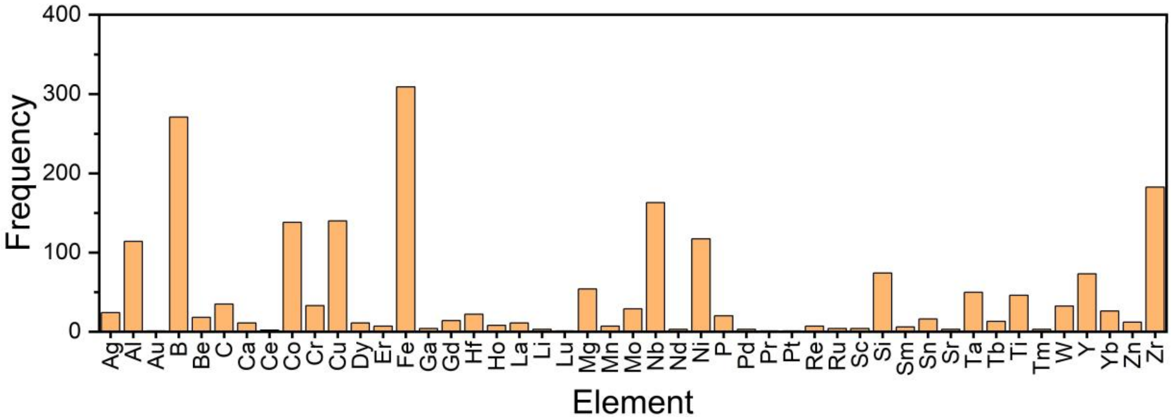

Supplementary Figure 1 – Frequency distribution of elements present in the input dataset. Source data are provided as a Source Data file.

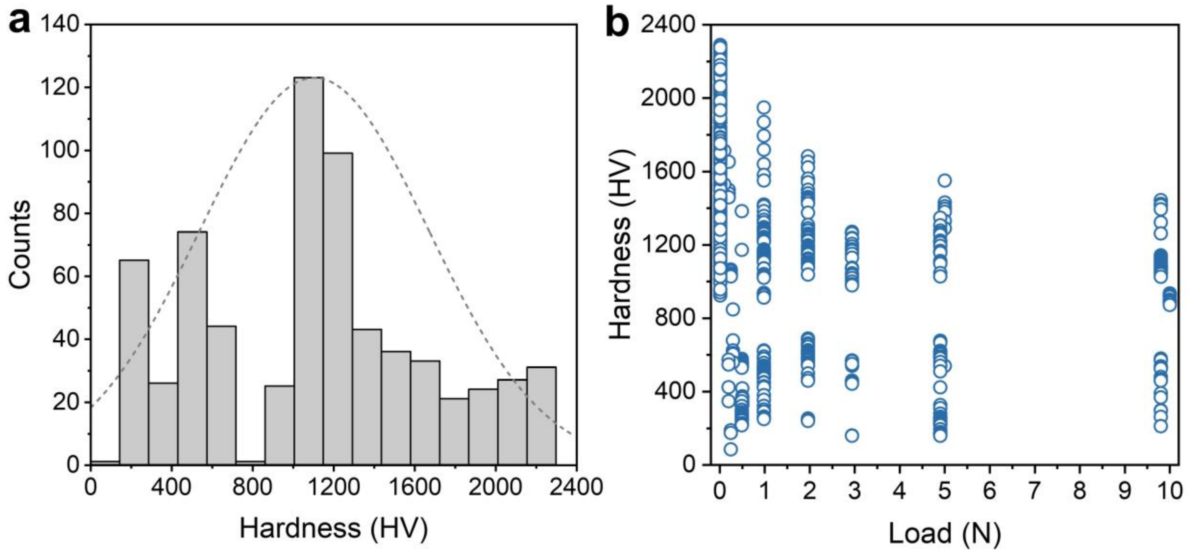

Supplementary Figure 2 – (a) Hardness (HV) distribution across the dataset, showing a multimodal pattern indicative of diverse alloy compositions and properties. (b) Scatter plot of hardness versus load, demonstrating the variation in hardness measurements with applied load. Source data are provided as a Source Data file.

Supplementary Note 2. Train-Test Split via Cluster-Stratified Sampling

To construct a chemically diverse and physically meaningful data split, we employed a cluster-aware stratified sampling strategy based solely on alloy compositions. First, compositional vectors were extracted from all 673 samples and clustered using K-means ($k = 3$), yielding chemically similar clusters. Importantly, this clustering was performed exclusively on elemental fractions, deliberately omitting mechanical properties (hardness) and experimental variables (load) to avoid label leakage. Next, we applied StratifiedShuffleSplit with a 20% test size, stratified by cluster labels. This ensured proportional representation of each compositional cluster in both the training and test subsets. Such an approach enhances model generalizability across underrepresented alloy chemistries and mitigates bias toward dominant composition families. To verify that compositional balance was retained, we computed the mean elemental fraction in both training and test sets (Supplementary Figure 3). The high agreement between the two distributions confirms that stratified cluster-based sampling successfully preserved the statistical and chemical diversity of the full dataset.

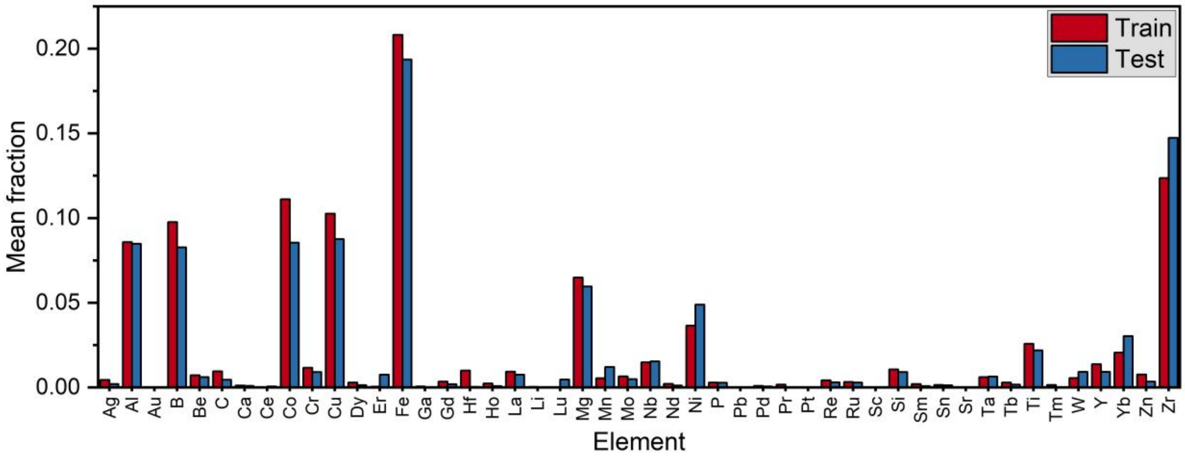

Supplementary Figure 3 – Element-wise mean atomic fraction in the training (538 alloys) and test (135 alloys) datasets. Source data are provided as a Source Data file.

Supplementary Note 3. Theoretical Foundations of the VIBANN Architecture

A concise reproducibility-level description of dataset splitting, model training, uncertainty estimation, inverse design, and baseline benchmarking is provided in the main Methods. This Supplementary Note provides extended theoretical formulation, algorithmic detail, and additional diagnostics.

The Information Bottleneck (IB) principle, originally proposed by Tishby et al., formalizes the idea that a good representation of input data should capture only the information necessary to predict the output, while discarding irrelevant details.[44] Originating from information theory, the VIB approach seeks to learn a compressed latent representation Z of the input data X that maximally preserves information relevant to the target property Y while systematically discarding superfluous details.[45] Unlike standard autoencoders or black-box neural regressors, VIB explicitly regularizes the latent space to produce a smooth, structured, and generative representation that is both interpretable and navigable.[44] In our setting, the input variable X represents the alloy composition and load, and the output variable Y is the predicted Vickers hardness. The goal is to learn a latent variable Z that compresses X as much as possible while preserving information relevant to Y.[45]

This is achieved by minimizing the functional:

$$\mathcal{L}_{IB} = I(Z;X) - \beta I(Z;Y)$$

where $I(\cdot;\cdot)$ denotes mutual information and β is a trade-off parameter controlling the strength of the bottleneck.[44] Since the exact computation of mutual information is intractable in deep neural networks, the Variational Information Bottleneck (VIB) approximates this objective using variational inference.[46] In the VIB approach, the encoder network maps each input x to a Gaussian distribution in latent space, defined by its mean $\mu(x)$ and standard deviation $\sigma(x)$. A latent vector z is sampled using the reparameterization trick:

$$z = \mu(x) + \sigma(x) \cdot \epsilon, \qquad \epsilon \sim N(0, I)$$

This stochastic representation is then passed to the decoder network, which predicts the output property. The VIB loss function is defined as:

$$\mathcal{L}_{VIB} = \mathbb{E}_{q(z|x)}[-log\, p(y|z))] + \beta D_{KL}(q(z|x)||p(z)),$$

The first term ensures predictive accuracy by minimizing the negative log-likelihood (typically mean squared error)[46] and the second term is the Kullback–Leibler divergence between the learned posterior $q(z|x)$ and a simple prior $p(z)$, usually taken as a standard

normal distribution. This KL regularization penalizes overly complex latent encodings and encourages compression.[47] The result is a model that learns to represent the compositional space in terms of a small number of abstract latent variables, each of which carries physically useful information relevant to the target property.

During training, we dynamically annealed the bottleneck weight β by progressively increasing it using a multiplicative factor, following a strategy similar to that proposed by Alemi et al.[45] This annealing emphasizes latent compression during the initial epochs and gradually shifts priority to predictive fidelity in later epochs, thereby stabilizing convergence. Recent applications of VIB in chemistry and materials informatics have demonstrated its utility in uncovering meaningful latent structure from noisy, high-dimensional inputs.[48-50] In our case, the VIB representation yielded interpretable latent dimensions, with only a subset significantly contributing to hardness prediction (see Figure 6a-c), indicating effective disentanglement and physical relevance.

To further enhance interpretability, we incorporate an **attention mechanism** that allows the model to selectively focus on the most important elements in the alloy composition. The attention mechanism in neural networks was originally developed in the context of sequence modeling[51] but has found broad applicability across domains, including materials science, due to its ability to focus on important features selectively.[52] In the VIBANN framework, attention is applied directly to the input composition vector to learn which elements in a multicomponent alloy contribute most significantly to the target property, such as hardness.

Formally, the input vector $x \in \mathbb{R}^d$ represents the atomic fractions of d different elements in the alloy. The attention mechanism computes a set of weights $a = [a_1, a_2, \dots, a_d]$, where each weight $a_i \in [0,1]$ reflects the importance of the element i for the prediction task. These weights are computed using a learnable function, often a shallow neural layer followed by a softmax operation to normalize the values[51]:

$$a = \mathrm{softmax}(W_a \cdot x + b_a)$$

where $W_a \in \mathbb{R}^{d \times d}$ and $b_a \in \mathbb{R}^d$ are trainable parameters, and the softmax function is defined as:

$$a_i = \frac{\exp(h_i)}{\sum_{j=1}^{d} \exp(h_j)}, \text{ where } h = W_a \cdot x + b_a$$

The result is a probability distribution over the input features, with elements receiving higher attention scores receiving stronger emphasis. These weights are then applied to the input via element-wise multiplication, producing an attended input vector:

$$x_{att} = a \odot x$$

where $\odot$ denotes the Hadamard (element-wise) product. The attended input x_{att} is passed downstream into the encoder of the VIB framework.

By assigning high weights to composition components important for hardness prediction and low or near-zero weights to irrelevant or redundant elements, the model effectively filters its input in a task-specific manner. Mathematically, the attention mechanism can be viewed as a feature-wise relevance map conditioned on the input. Unlike static feature selection techniques, attention is fully differentiable and adaptable: it automatically tunes its focus as the model sees new data, while simultaneously providing interpretable weightings that can be visualized and analyzed.[53] Importantly, the attention scores are learned jointly with the rest of the model through backpropagation, meaning they adapt dynamically to the training data. This attention mechanism offers two levels of interpretability. First, globally, the average attention weights over the training dataset reveal which elements consistently influence hardness. Second, contextually, for a given alloy composition, the attention scores dynamically adjust to emphasize contextually important elements based on local compositional interactions. In practice, this interpretability guided our alloy design process. As shown in Figure 6c, the attention module consistently highlighted elements such as B, Nb, Co, and W, which are known to contribute to high hardness. These insights directly informed the inverse design stage, where we prioritized compositions enriched with such elements.

By combining VIB and attention, the VIBANN model not only achieves high predictive performance but also yields physically structured latent representations and element-wise relevance maps. This dual interpretability bridges deep learning with metallurgical insight, enabling both understanding and actionable inverse design of new multicomponent metallic glasses.

S3.1 Regularization Mechanisms in VIBANN:

To prevent overfitting and ensure robust generalization, the VIBANN model integrates multiple synergistic regularization strategies, summarized in the table below.

**Type**	**Implementation**	**Purpose**
Dropout	Composition and load branches (dropout rates tuned via Bayesian optimization)	Prevents co-adaptation; supports epistemic uncertainty estimation
Batch Normalization	Post-Dense layers in both input branches	Normalizes activations and provides implicit regularization
VIB KL Divergence Loss	Explicit KL term with adaptive β annealing	Encourages compressed, task-relevant latent representations
Adaptive β Tuning	KL-weight β adjusted per epoch based on KL divergence magnitude	Balances latent compactness vs predictive performance
Attention Mechanism	Feature-wise attention is applied before the VIB layer	Reduces effective input dimensionality; promotes sparsity
Bayesian Hyperparameter Optimization	Optuna-based search over latent dimensions and dropout	Ensures model complexity is data-optimal, not arbitrarily chosen
Stratified K-Fold CV	Cluster-based stratification with equal compositional representation	Validates robustness across diverse alloy families

Algorithm S1. Prediction model training

Inputs:

- Training dataset $\mathcal{D} = \left\{\left(x_{comp}^{(i)}, x_{load}^{(i)}, y^{(i)}, w^{(i)}\right)\right\}_{i=1}^{N}$, where $x_{comp} \in \mathbb{R}^d$ (composition features), $x_{load} \in \mathbb{R}$ (load), y (hardness), and w (sample weights).
- Pre-processing/scalers for x_{load} and y; hyperparameter search space.

Outputs:

Trained surrogate final_model (includes attention, VIB latent, Monte-Carlo Dropout), fitted scaler_load_final, output standardization constants (μ_y,σ_y) stored as y_mu_final, y_sd_final, attention scores; Comp_Attention, Load_Attention.

Procedure:

1. **Preprocessing**
   A. Split $\mathcal{D}$ into training/validation/test (Stratified Splitting to preserve distribution among the subsets).
   B. Fit the load scaler on training loads; transform all loads:
   $$x_{load,scaled} \leftarrow \text{scaler\_load}(x_{load}).$$
   C. Standardize targets to scaled space:
   $$y_{scaled} \leftarrow \frac{(y-\mu_y)}{\sigma_y}.$$

2. **Define the architecture** (Attention → trunk → VIB → prediction head)
   A. **Inputs:** (x_{comp}, $x_{load,scaled}$).
   B. **Composition attention module:** compute attention-weighted composition representation $\tilde{x}_{comp}$ using the attention subnetwork in final_model.
   C. **Feature trunk:** apply dense transformations with BatchNorm and MCDropout layers (custom dropout that can be toggled during inference).
   D. **VIB layer (vib_layer):**
      - compute latent parameters ($\mu_z, \log\sigma_z^2$) and sample
      $$z = \mu_z + \sigma_z \odot \epsilon, \qquad \epsilon \sim \mathcal{N}(0, I),$$
      - add the KL term $\text{KL}(q(z|\cdot)\|p(z))$ to the total loss via the layer's internal add_loss.
   E. **Prediction head:** output scaled hardness $\hat{y}_{scaled}$ via the layer named "Output_Layer".
   F. **Attention Scores:** exported attention tensors (trained implicitly; zero-loss); Comp_Attention, Load_Attention

3. **Training objective**
   A. Weighted regression loss in scaled space:
   $$\mathcal{L}_{reg} = \sum_{i=1}^{N} w^{(i)}\left(\hat{y}_{scaled}^{(i)} - y_{scaled}^{(i)}\right)^2.$$
   B. Total loss:
   $$\mathcal{L} = \mathcal{L}_{reg} + \lambda_{recon} D_{KL}(x\|\hat{x}) + \beta(t)\frac{1}{d}KL(q(z|x)\|p(z)),$$
   where $\beta(t)$ follows the schedule/controller implemented in the code.

4. **Hyperparameter selection (Bayesian Optimization)**
    A. For each hyperparameter trial: instantiate the model, train with early stopping/checkpointing, evaluate on validation.
    B. Select the best trial and retrain/refit the final model accordingly.

5. **Batch Normalization-safe Monte-Carlo dropout configuration (for uncertainty quantification)**
    - Enforce BatchNorm freezing by evaluating with training=False.
    - Enable epistemic uncertainty only by toggling MCDropout via the mc_active flag.

Algorithm S2. MC-dropout epistemic uncertainty for the trained Attention-VIB surrogate

Goal:

Approximate the predictive distribution of hardness at fixed load using Monte Carlo dropout while keeping BatchNorm frozen, and compute μ_{HV}, σ_{HV} and risk-aware LCB.

Inputs:

- Trained model final_model with output Output_Layer (or first element of list/tuple output).
- Candidate compositions $X_{comp} \in \mathbb{R}^{N\times d}$.
- Design load L (raw, in N) and fitted load scaler scaler_load_final.
- Output scaling constants (μ_y, σ_y) used during training.
- MC sample count S, confidence parameter z, batch size B.
- Base random seed seed_base.

Outputs:

- $\mu_{HV} \in \mathbb{R}^N$, $\sigma_{HV} \in \mathbb{R}^N$, $LCB_{HV} \in \mathbb{R}^N$.
- Quantiles q_p (e.g., $p = 0.10, 0.80$).
- The full MC sample matrix $HV^{(s)} \in \mathbb{R}^N$ for $s = 1 \dots S$.

Procedure:

1. **Pre-process inputs (conditioning variables)**
   A. Project compositions onto the simplex (nonnegative, sum-to-one):
   $$X_{comp} \leftarrow \prod_\Delta(X_{comp}).$$
   B. Form a raw load vector $X_{load,raw} \leftarrow L \cdot 1_{N\times 1}$.
   C. Scale load using the training scaler:
   $X_{load,raw} \leftarrow \text{scaler}_{\text{load}_{\text{final}}}.\text{transform}(X_{load,raw})$.

2. **Configure BN-safe MC-dropout (epistemic only)**
   A. Enforce BatchNorm freezing by always evaluating the model with training=False.
   B. Enable stochasticity only in custom dropout layers:
   - Traverse all sublayers of final_model.
   - For each layer m: if m is of class "MCDropout" and has attribute mc_active, set: $\text{m.mc\_active} \leftarrow \text{True}$.
   - All other layers (including BatchNorm) remain deterministic under training=False.

3. **Verify MC-dropout is active under training=False (required diagnostic)**
   A. For a small test batch $(X_{comp}^{test}, X_{load,scaled}^{test})$, run S_{check} stochastic forward passes with distinct seeds.
   B. Compute the mean per-point standard deviation:
   $$\bar{s} \leftarrow mean_i\left(std_s(\hat{y}_{scaled,i}^{(s)})\right).$$
   C. If $\bar{s} \le \varepsilon$, abort: MC-dropout is not active (uncertainty estimates would be invalid).

4. Generate an independent seed stream (reproducible, uncorrelated draws)

A. Create a seed sequence with SeedSequence(seed_base) and generate S integer seeds:

$$\{seed_s\}_{s=1}^{S}.$$

This prevents correlated MC samples while preserving full reproducibility.

5. Monte Carlo dropout sampling (BN frozen)

A. Initialize samples_scaled with shape (S,N).

B. For each draw s = 1, …, S:

- A. Set the global RNG state using seed_s.
- B. Iterate over mini-batches of $(X_{comp}, X_{load,scaled})$ of size B.
- C. Evaluate:
  $\hat{y}_{\text{scaled}}^{(s)} \leftarrow \text{final\_model}([X_{comp}, X_{load,scaled}], \text{training} = \text{False})$.
- D. Extract the scalar prediction vector robustly:
  - If output is a dict: use Output_Layer.
  - If output is a list/tuple: use the first element.
  - Else: use the output tensor directly.
- E. Store $\hat{y}_{\text{scaled}}^{(s)}$ in samples_scaled[s,:].

C. Disable MC-dropout (set mc_active=False) after sampling.

6. Convert to hardness and summarize epistemic uncertainty

A. Convert scaled predictions to hardness: $HV^{(s)} \leftarrow \hat{y}_{scaled}^{(s)}\sigma_y + \mu_y$.

B. Compute predictive mean: $\mu_{HV} \leftarrow \frac{1}{S}\sum_{s=1}^{S} HV^{(s)}$.

C. Compute epistemic standard deviation: $\sigma_{HV} \leftarrow \sqrt{\frac{1}{S}\sum_{s=1}^{S}(HV^{(s)} - \mu_{HV})^2}$.

D. Compute risk-aware lower confidence bound (LCB): $\text{LCB}_{HV} \leftarrow \mu_{HV} - z\sigma_{HV}$.

E. Compute quantiles q_p over $\{HV^{(s)}\}$ (e.g., p = 0.10, 0.80).

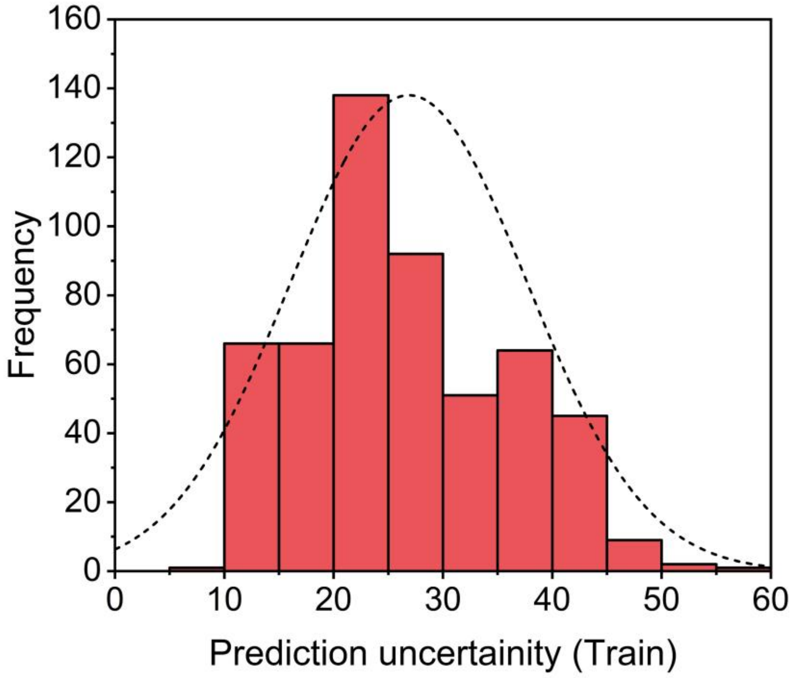

Supplementary Figure 4 – Histogram of prediction uncertainty for the training set. The distribution shows varying model confidence across samples, with a fitted curve indicating a moderately skewed spread of uncertainties. Source data are provided as a Source Data file.

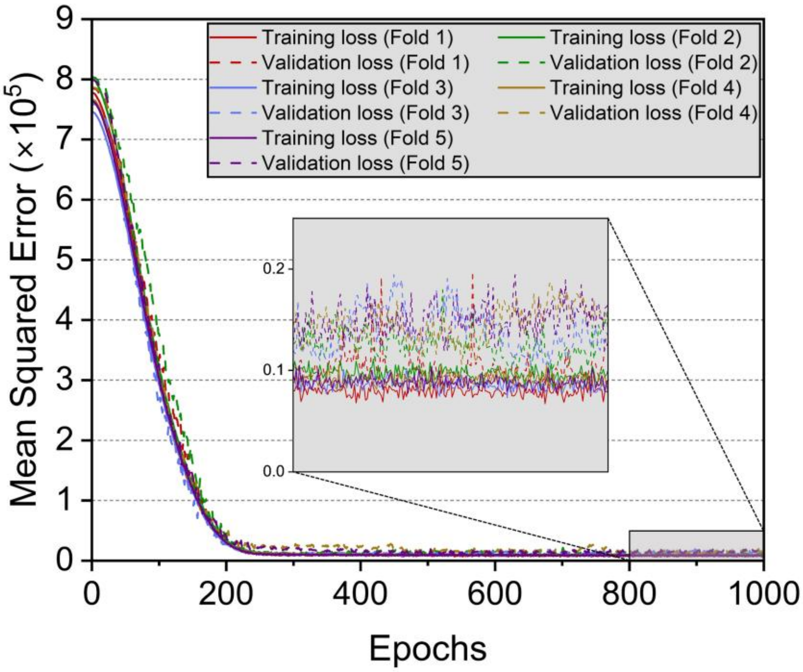

Supplementary Figure 5 – Training and validation loss for the VIBANN model across five folds. Mean squared error (MSE) decreases consistently across epochs for all folds, indicating stable convergence. The inset highlights variations in validation loss, reflecting fold-specific generalization behavior. Source data are provided as a Source Data file.

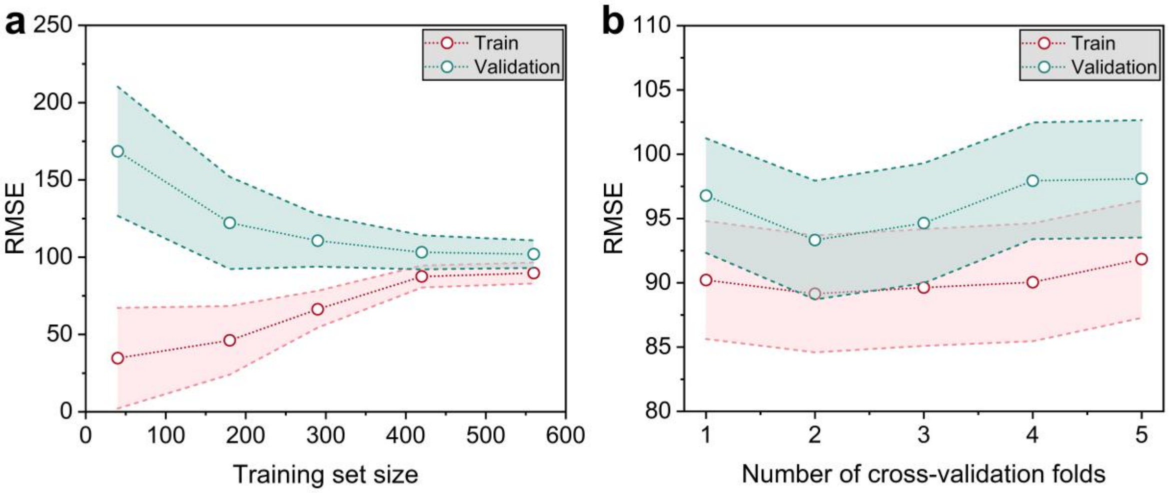

Supplementary Figure 6 – (a) RMSE as a function of training set size showing improved generalization with larger datasets. Points denote mean RMSE across n = 5 cross-validation folds, and shaded bands denote ±1 standard deviation (s.d.) across the five folds. (b) RMSE variation with the number of cross-validation folds, indicating stable performance beyond 3-fold validation. For each k-fold setting, points denote mean RMSE across n = k folds (k = 1, 2, 3, 4, and 5, respectively), and shaded bands denote ±1 s.d. across those folds. Source data are provided as a Source Data file.

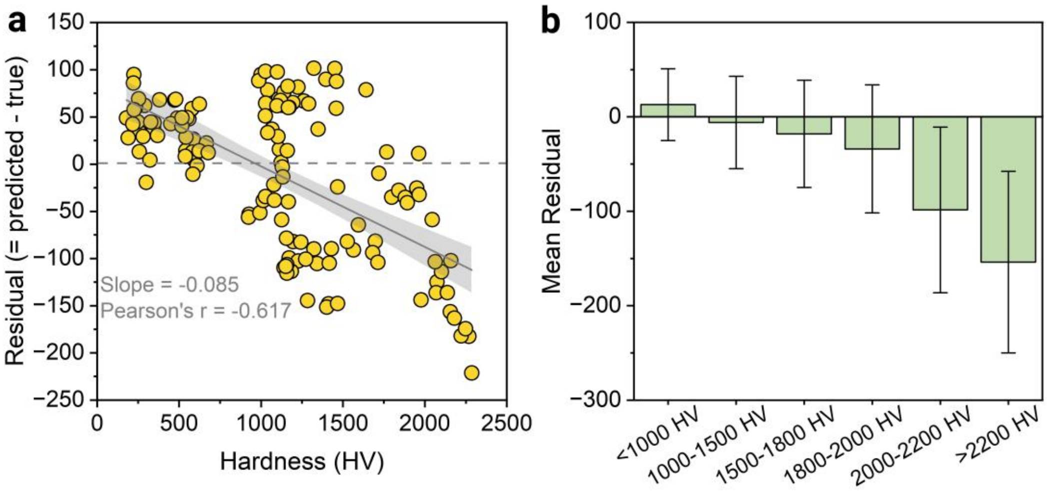

Supplementary Figure 7 – Residual analysis of VIBANN predictions across the hardness range. (a) Residuals, defined as predicted minus true hardness, are plotted against true hardness for the held-out test set (n = 135 observations), where one observation corresponds to one composition–load–hardness entry. The linear fit (grey solid line) shows a progressive negative residual trend with increasing hardness, indicating increasingly conservative predictions in the upper-hardness regime. The grey shaded band denotes the 95% confidence interval of the fit. (b) Mean signed residual in bins of true hardness. Bars denote the mean residual within each hardness bin and error bars denote ±1 standard error of the mean (s.e.m.) from the observations in that bin. The exact sample sizes are n = 32 for <1000 HV, 43 for 1000–1500 HV, 28 for 1500–1800 HV, 16 for 1800–2000 HV, 10 for 2000–2200 HV, and 6 for >2200 HV, respectively. The binned analysis shows that the predictive mean is near unbiased at low hardness but becomes progressively more negative in the higher-hardness bins, consistent with increasing underprediction in the sparsely populated tail of the training distribution. Source data are provided as a Source Data file.

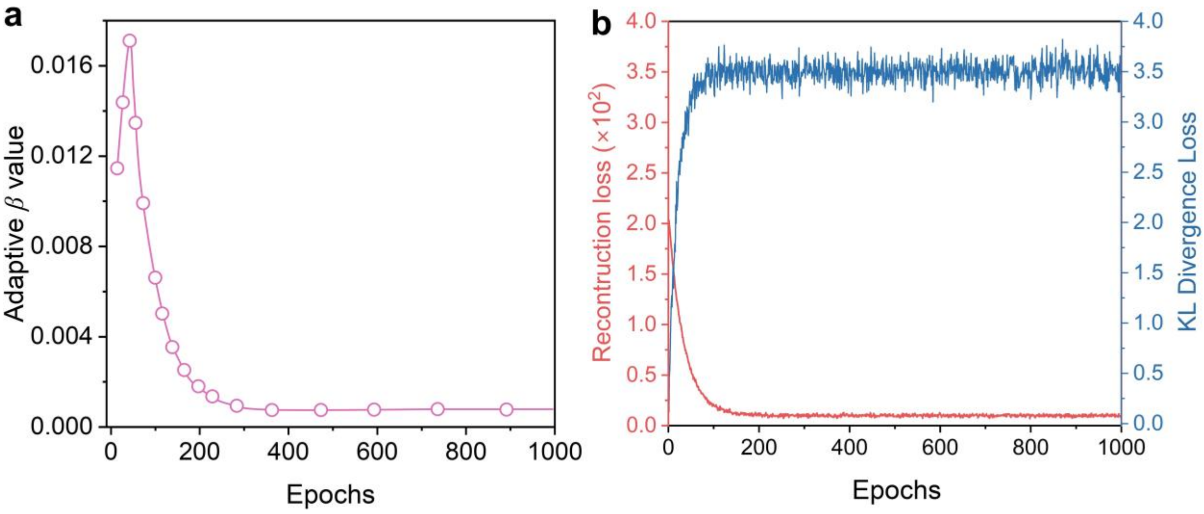

Supplementary Figure 8 – (a) Adaptive β value evolution across epochs during training of the VIBANN model. The β parameter balances the trade-off between reconstruction accuracy and latent space regularization. (b) KL divergence and reconstruction loss evolution across epochs, demonstrating effective optimization of the latent space regularization. The significant drop in the reconstruction loss during the first 150 epochs indicates efficient balancing of reconstruction accuracy and latent space compression in the VIB framework. Source data are provided as a Source Data file.

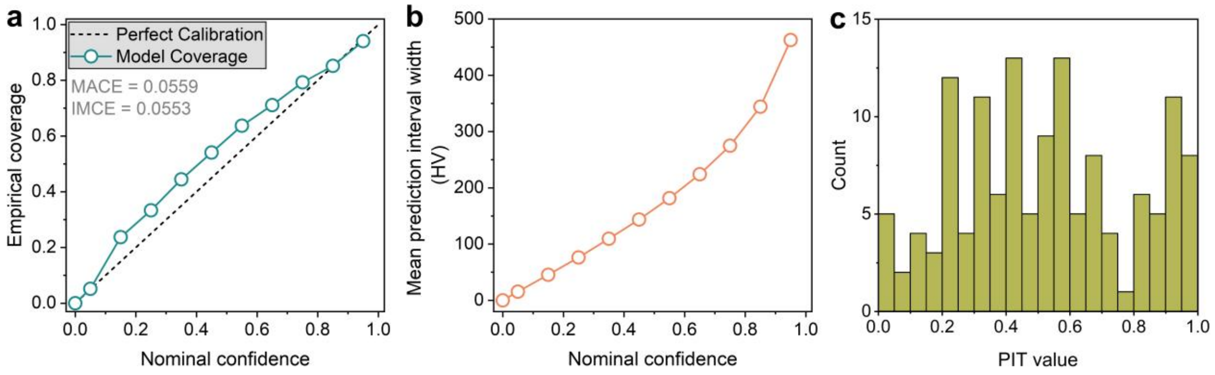

Supplementary Figure 9 – Calibration diagnostics for VIBANN predictive uncertainty. (a) Reliability diagram comparing empirical coverage of the Monte Carlo dropout predictive intervals to the nominal confidence level. The dashed line indicates perfect calibration, and the markers show the measured coverage across confidence bins, with summary calibration errors reported as Mean Absolute Calibration Error (MACE) and Integrated Mean Calibration Error (IMCE). (b) Mean predictive interval width as a function of nominal confidence, showing the expected monotonic increase in interval width for higher confidence levels. (c) Probability integral transform (PIT) histogram for the standardized residual distribution, where an approximately uniform distribution indicates well-calibrated predictive uncertainty. Source data are provided as a Source Data file.

Supplementary Note 4. Benchmarking Predictive Models for Hardness in MMGs

Supplementary Figures 10-12 present a comprehensive benchmarking analysis of our proposed VIBANN framework against six widely used machine learning regressors: Lasso regression, Ridge regression, k-Nearest Neighbors (KNN), Random Forest (RF), Gradient Boosting (GB), and a Multilayer Perceptron (MLP). Each model was trained to predict the Vickers hardness (HV) of MMGs using alloy compositions and applied loads as input features. Model performance is quantitatively compared in Supplementary Figure 10 using the coefficient of determination (R^2) and root mean squared error (RMSE), while learning curves for each method are shown in Supplementary Figure 11. The linear models, Lasso and Ridge, exhibited moderate predictive capacity with R^2 scores of 0.753 and 0.751, respectively. Their relatively high RMSEs (~222 HV) highlight their limited ability to capture the nonlinear, high-order feature interactions inherent to disordered MMG systems. These models rely on additive, low-complexity assumptions and lack the flexibility to encode interdependencies between composition, load, and hardness: factors that are inherently entangled through underlying metallurgical phenomena such as short-range ordering, bond stiffness variation, and load-induced deformation mechanisms.

Nonlinear ensemble models like RF (R^2 = 0.852, RMSE = 172.3 HV) and GB (R^2 = 0.866, RMSE = 163.8 HV) achieved some improvements by exploiting feature interactions and hierarchical partitioning. Gradient Boosting slightly outperformed RF, likely due to its sequential bias-reduction strategy, which enables more efficient modeling of subtle interactions. However, these tree-based methods offer limited insight into extrapolation behavior or uncertainty quantification, which are critical in materials design tasks. The KNN model (R^2 = 0.821, RMSE = 189.4 HV) also shows unsatisfactory performance as it is inherently non-parametric and highly dependent on local data density. Its ability to interpolate within dense regions is strong, but it performs poorly in sparse regions, rendering it unsuitable for extrapolating to novel or out-of-distribution compositions, a fundamental requirement in materials discovery workflows. The MLP model yielded the strongest results among conventional deep learning architectures (R^2 = 0.886, RMSE = 143.1 HV), benefitting from its ability to approximate complex functions. However, it lacks the regularization and interpretability mechanisms required for physically meaningful, uncertainty-aware predictions. Crucially, the VIBANN model significantly outperformed all baselines, achieving the highest R^2 (0.943) and lowest RMSE (101.4 HV) on the test set (see Supplementary Figure 10). Although GB is a

strong nonparametric baseline, its test error remains ~163.8 HV higher than that of VIBANN, and its R^2 is ~0.08 lower (Supplementary Figures 10e, 12). The error gap widens in the high-hardness tail, which is most relevant for discovery, where VIBANN reduces the mean absolute error by ~40% relative to GB.

Beyond predictive accuracy, VIBANN integrates variational inference and information bottleneck principles to filter irrelevant features and retain only the most informative latent representations. This mechanism facilitates generalization and guards against overfitting, particularly in high-dimensional composition–property spaces. Moreover, through Monte Carlo dropout and variational posterior sampling, VIBANN provides epistemic uncertainty estimates, a key capability absent in most conventional models. This is particularly valuable in materials discovery pipelines, where model confidence directly informs experimental prioritization and risk management. The model's latent space encodes chemically meaningful relationships, further enhanced by the self-attention mechanism, which selectively weights input features, aiding interpretation and guiding alloy design decisions. Finally, unlike GB, VIBANN provides calibrated epistemic uncertainty and element-resolved attention maps that we used to prioritize alloy chemistries for experimental synthesis; these interpretability and risk-aware features were essential to the successful discovery of the exceptionally hard MMGs reported in the main text.

Collectively, the benchmarking analysis validates VIBANN as a state-of-the-art framework for data-efficient, interpretable, and uncertainty-aware prediction of mechanical properties in complex, high-dimensional alloy systems. It aligns with the broader goals of materials informatics: not only to predict properties but also to enable the rational, explainable design of new materials with targeted functionalities.

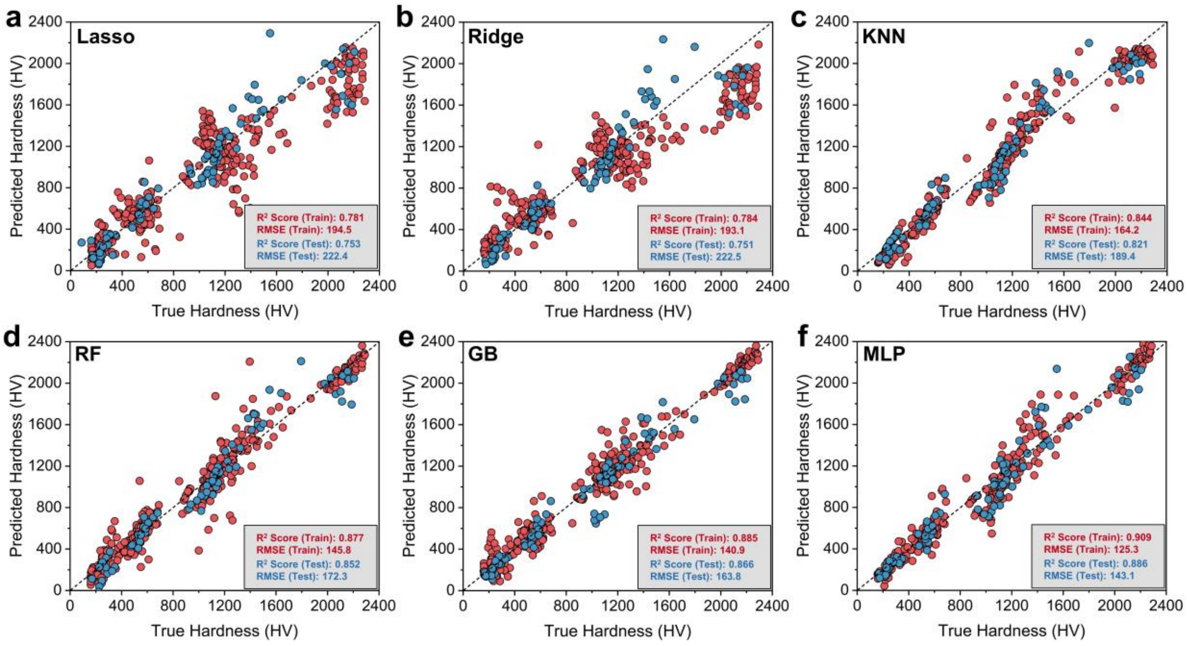

Supplementary Figure 10 – Performance comparison of various machine learning models in predicting the hardness (HV) of multicomponent metallic glasses. Predicted vs. actual hardness plots for (a) Lasso regression, (b) Ridge regression, (c) Random Forest (RF), (d) k-Nearest Neighbors (KNN), and (e) Gradient Boosting (GB); (f) Multilayer Perceptron (MLP) models. Source data are provided as a Source Data file.

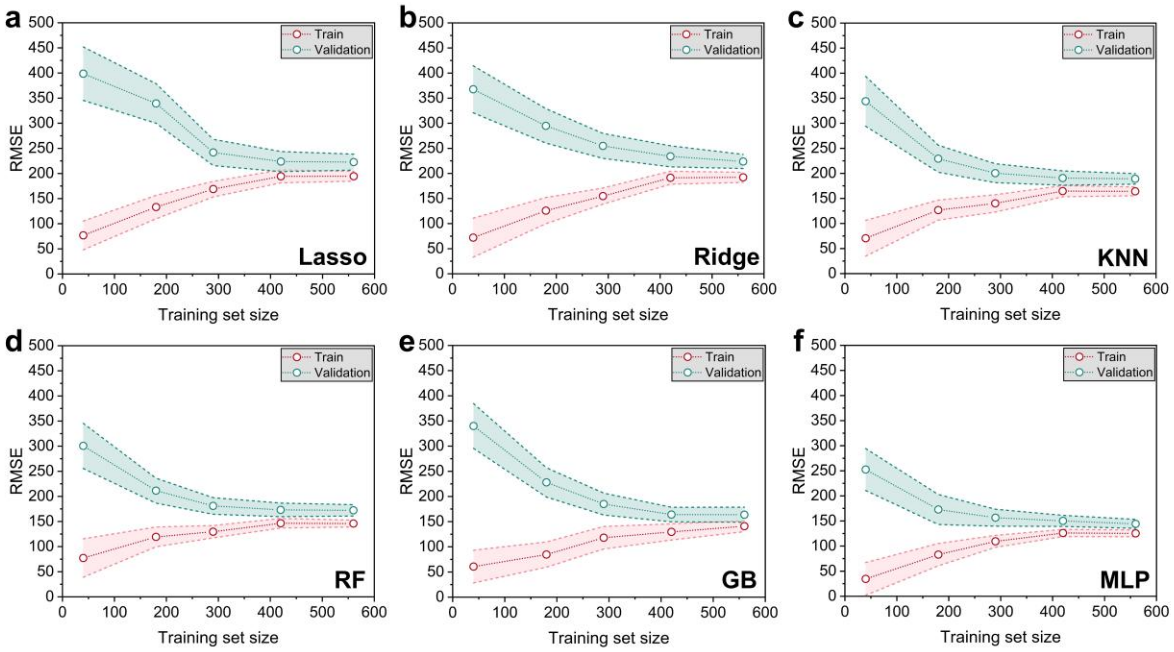

Supplementary Figure 11 – Effect of training set size on model performance across different algorithms. RMSE trends for training and validation sets are shown for (a) Lasso, (b) Ridge, (c) KNN, (d) Random Forest (RF), (e) Gradient Boosting (GB), and (f) Multilayer Perceptron (MLP). For each algorithm and training-set size, points denote mean RMSE across n = 5 cross-validation folds, and shaded bands denote ±1 s.d. across the five folds. Source data are provided as a Source Data file.

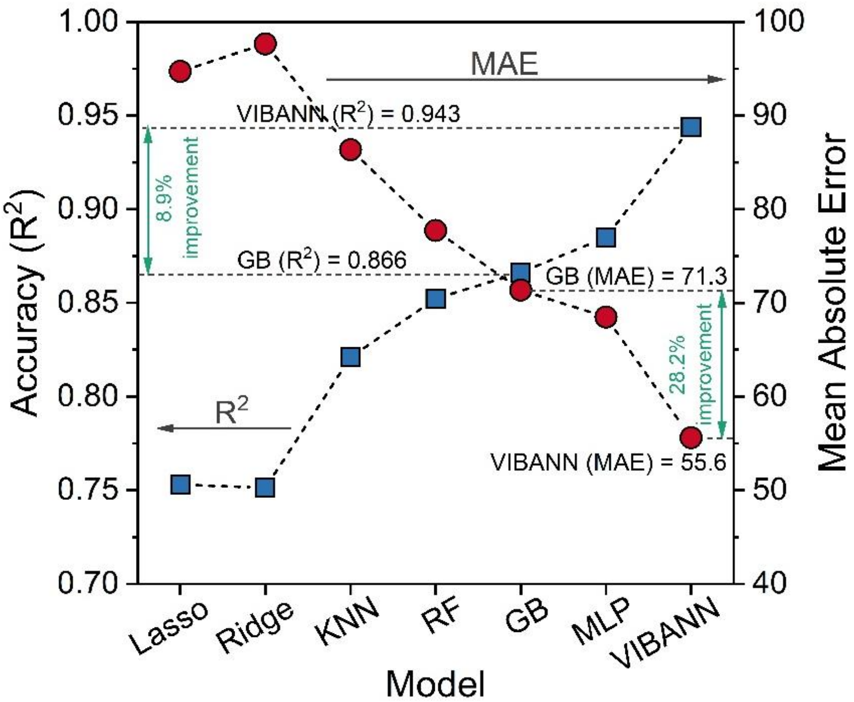

Supplementary Figure 12 – Summary plot comparing accuracy (R^2) and mean absolute error (MAE) for all models, with the VIBANN model achieving the best performance among the models. Source data are provided as a Source Data file.

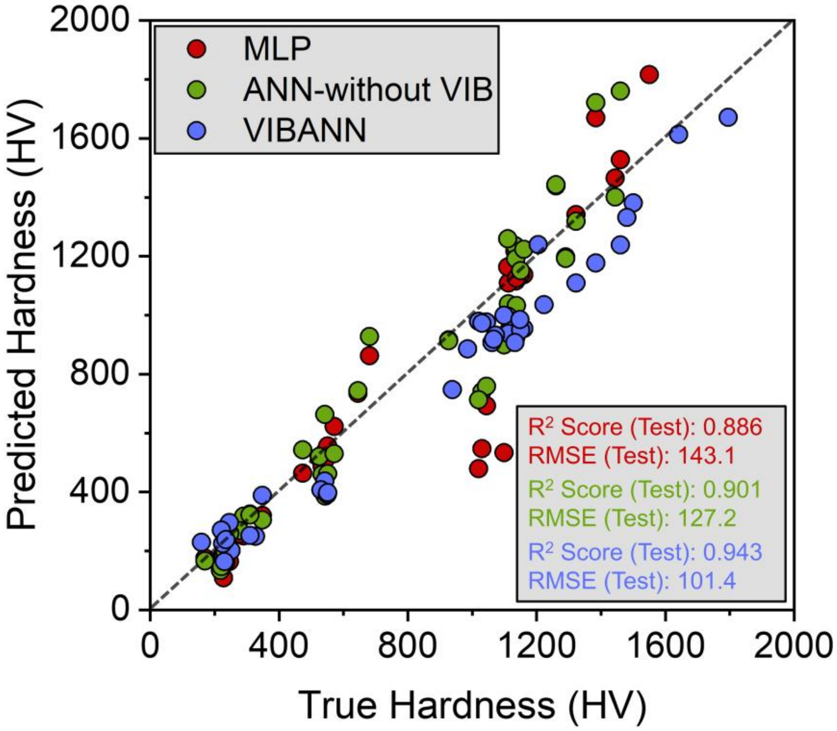

Supplementary Figure 13 – Comparison of predicted versus true hardness values for different baseline models. The scatter plot compares MLP, ANN without VIB, and VIBANN predictions on the test set. VIBANN shows the highest accuracy with the lowest RMSE and highest R^2, indicating superior generalization performance. Source data are provided as a Source Data file.

Supplementary Note 5. Compositional Clustering and Visualization of Chemical Subdomains

To enable chemically informed stratification and enhance interpretability in downstream machine learning tasks, we performed unsupervised clustering on the normalized compositional vectors of all MMGs. The objective was to uncover latent chemical subdomains in the composition space and utilize them for cluster-aware model training, feature attribution, and guided inverse design.

The optimal number of clusters k was determined using the Elbow Method, based on K-means clustering applied directly to the composition space. We computed the within-cluster sum of squares (WCSS) for $k = 1$ to 10, which quantifies intra-cluster compactness. As shown in Supplementary Figure 14, WCSS drops sharply until $k = 3$, after which the marginal gain diminishes, indicating an inflection point at $k = 3$. This point corresponds to the balance between under- and over-partitioning, suggesting that a three-cluster structure effectively captures the dominant modes of compositional variability without introducing spurious granularity. The clustering was conducted exclusively on the normalized molar fraction vectors of constituent elements. No information about hardness or experimental variables (e.g., applied load) was included at this stage, thereby eliminating the risk of target leakage. We also verified that clustering on raw (unnormalized) vectors led to distorted partitions biased by dominant elements, underscoring the need for standardized input. This chemically grounded clustering reveals non-random, physically meaningful groupings within the alloy dataset.

To further assess the structure and separability of the identified clusters, we used t-distributed Stochastic Neighbor Embedding (t-SNE) to project the high-dimensional composition vectors onto a two-dimensional manifold. This nonlinear technique preserves local similarities and enables intuitive visualization of complex chemical relationships. As shown in Supplementary Figure 15, the t-SNE projection reveals three partially distinct clusters, consistent with the K-means results. Each data point corresponds to a unique MMG alloy and is color-coded by its assigned cluster label.

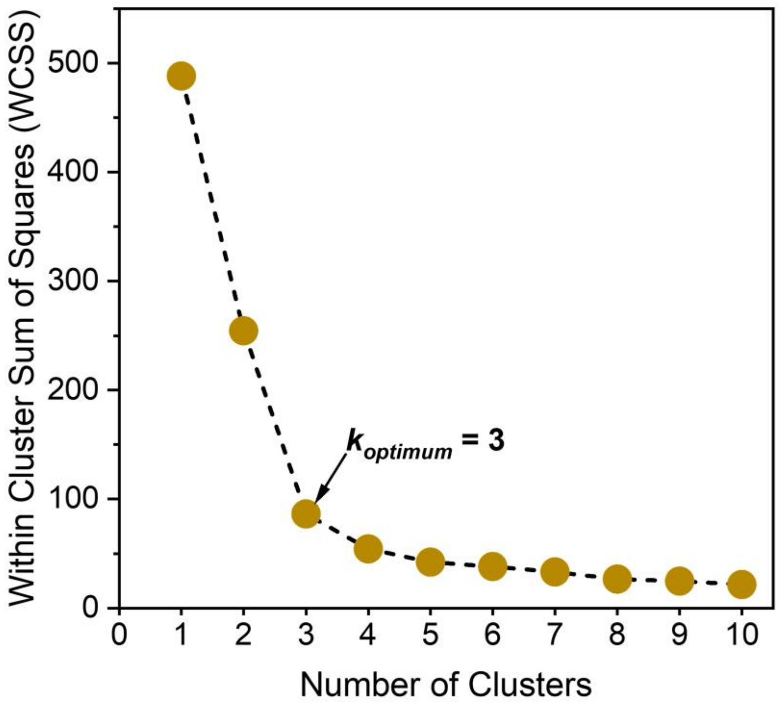

Supplementary Figure 14 – Elbow plot showing the within-cluster sum of squares (WCSS) as a function of the number of clusters for K-means clustering. The optimum number of clusters ($k_{optimum}$ = 3) is identified at the "elbow" point, where the rate of decrease in WCSS slows, indicating the most appropriate number of clusters. Source data are provided as a Source Data file.

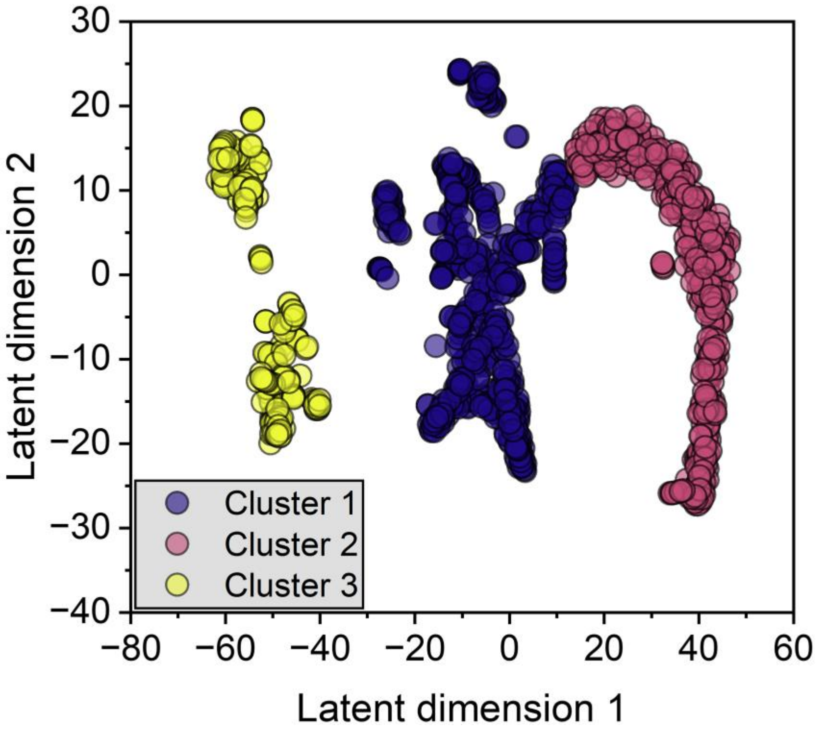

Supplementary Figure 15 – t-SNE projection of the VIB latent space, colored by cluster assignment into three distinct groups (Cluster 1: violet, Cluster 2: rose, Cluster 3: yellow). Each point represents an alloy sample embedded in a 2D latent space, revealing well-separated and compact clusters that reflect distinct structural or compositional characteristics captured by the VIBANN model. Source data are provided as a Source Data file.

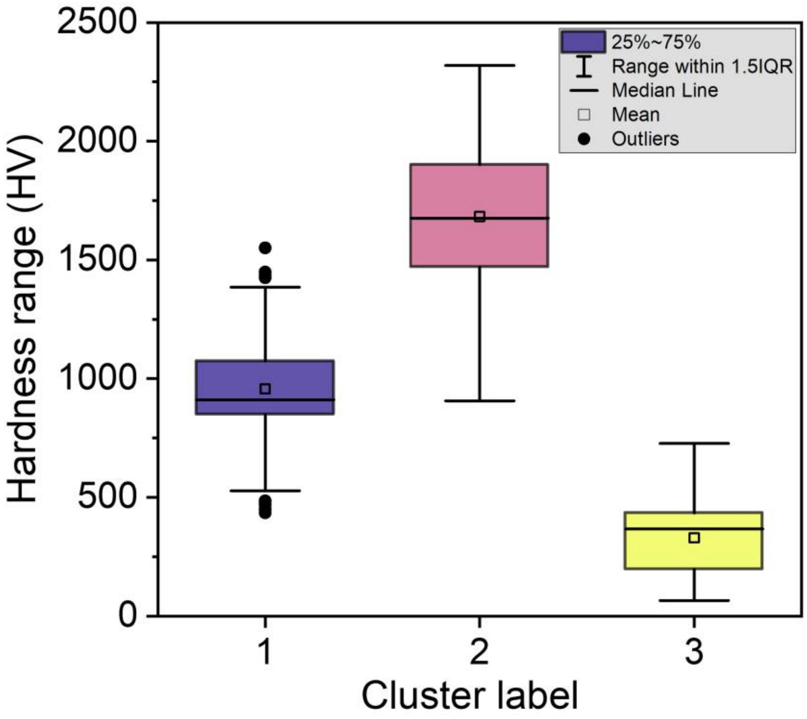

Supplementary Figure 16 – Boxplot illustrating the distribution of measured hardness (HV) values for the three distinct clusters. The horizontal line within each box denotes the median hardness, while the upper and lower edges of the box represent the 75th (Q3) and 25th (Q1) percentiles, respectively. The whiskers extend to 1.5 times the interquartile range (IQR) beyond Q1 and Q3, and any points lying outside these whiskers are plotted as outliers. Source data are provided as a Source Data file.

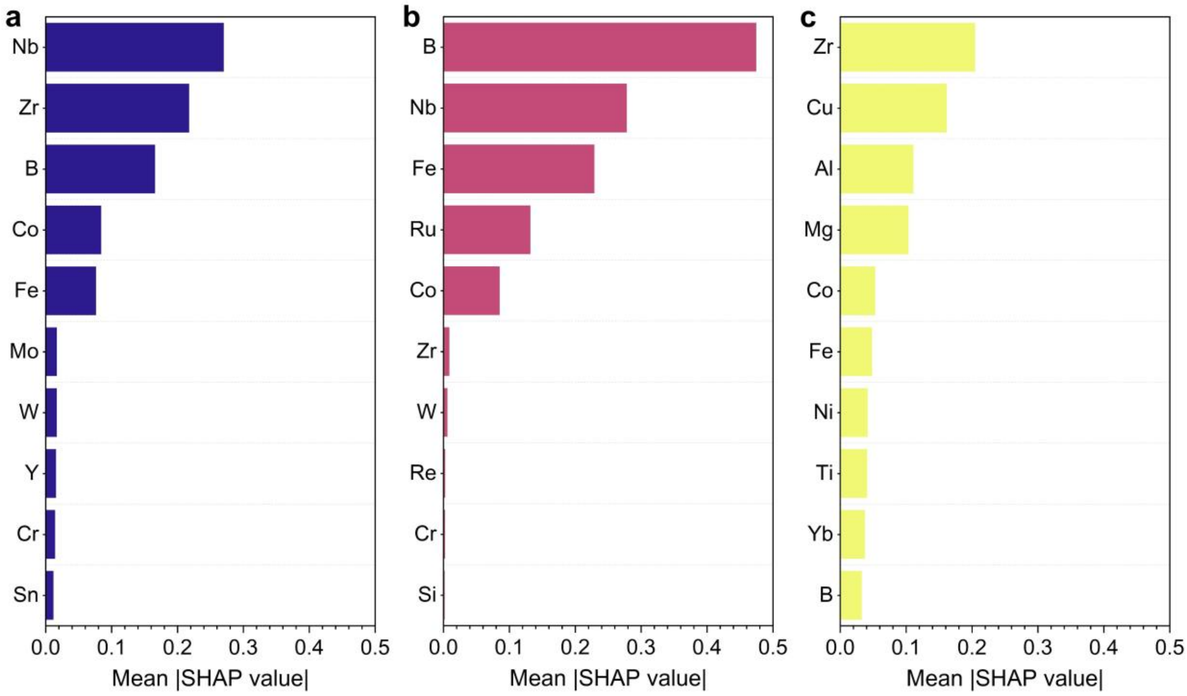

Supplementary Figure 17 – Permutation-based feature importance analysis for different clusters. Mean absolute SHAP values for (a) Cluster 1, (b) Cluster 2, and (c) Cluster 3 highlight the relative contribution of 10 most important elements to hardness prediction. Source data are provided as a Source Data file.

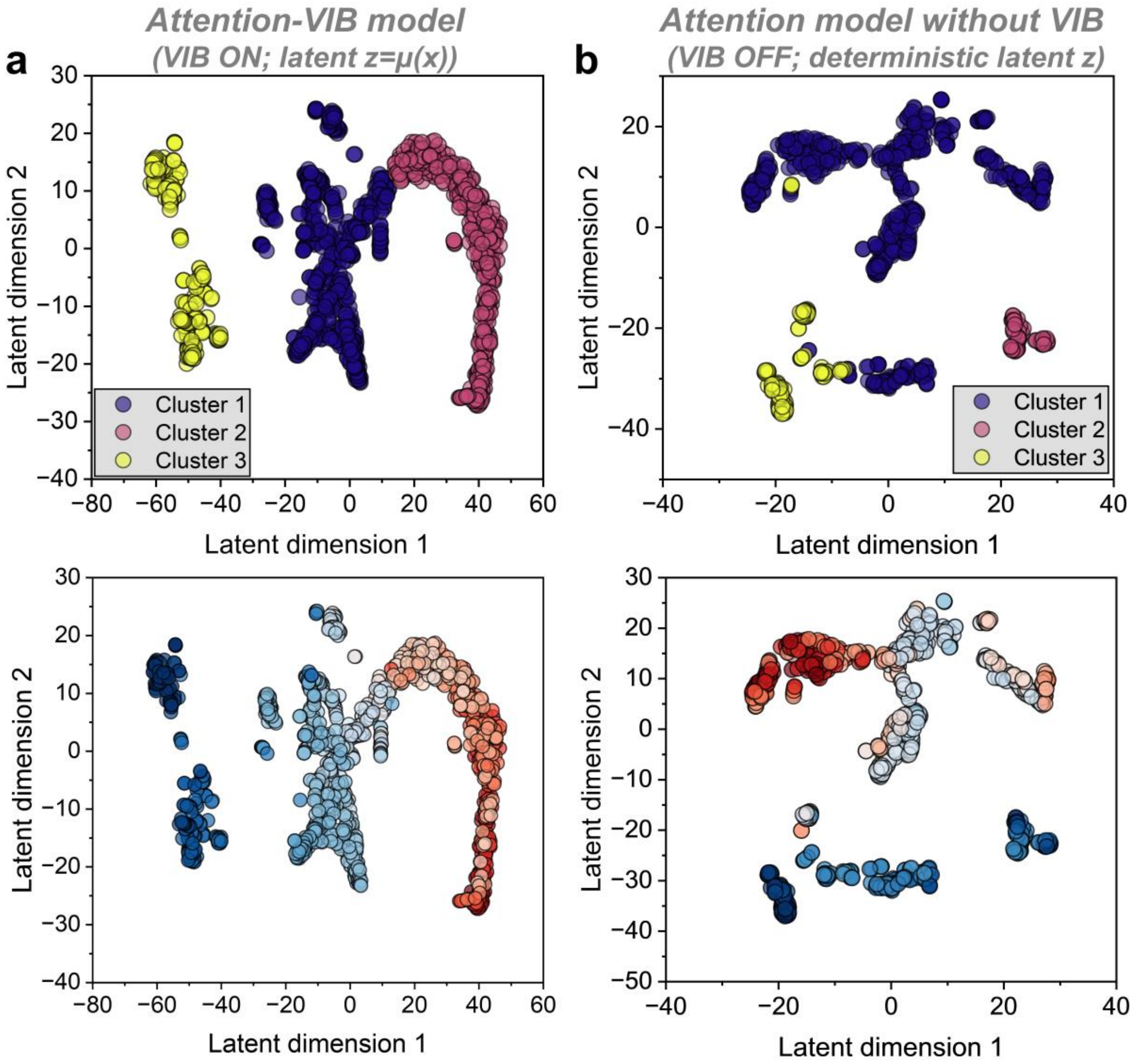

Supplementary Figure 18 – Effect of the variational information bottleneck (VIB) on latent-space structure and property organization. Comparative latent-space maps for the same attention-based predictor trained under identical data splits, latent dimensionality, and optimization settings, differing only by the inclusion of the VIB bottleneck. **(a)** Left (VIB ON): latent coordinates correspond to the posterior mean $z = \mu(x)$. **(b)** Right (VIB OFF): the VIB is replaced by a deterministic bottleneck of the same dimensionality. Top row: latent points colored by cluster assignment. Bottom row: the same latent points colored by hardness. Inclusion of VIB yields a more coherent and connected latent organization with clearer separation between clusters and smoother stratification of hardness across latent directions, whereas removal of VIB leads to a more fragmented latent arrangement with disconnected components and less continuous property gradients. Source data are provided as a Source Data file.

Supplementary Note 6. Assessment of latent-space navigability

To complement the qualitative latent-space maps in Supplementary Figure 18 with quantitative evidence for "navigability", we evaluated latent-space connectivity and local smoothness using graph- and slope-based diagnostics computed on the same latent coordinates shown in Supplementary Figure 18. For each datapoint, we identified its k=10 nearest neighbors in the 2D latent space (Euclidean metric) and constructed a directed k-nearest-neighbor (kNN) graph by connecting each point to its neighbors. We then quantified connectivity by calculating (i) the number of connected components and (ii) the fraction of points contained in the largest connected component, which distinguishes a single traversable latent region from a representation fragmented into disconnected islands at the same neighborhood scale. With VIB enabled, the kNN graph was largely connected, comprising 3 components, with ~0.952 of the points in the largest component, consistent with a single dominant traversable region and a small number of isolated islands. In contrast, the deterministic bottleneck (VIB OFF) produced a more fragmented topology with 8 connected components and a reduced largest-component fraction of ~0.691, indicating multiple disconnected islands under identical graph construction.

We further quantified local smoothness of the hardness landscape by computing a Lipschitz-proxy slope along each kNN edge, defined as $|\Delta HV| / \|\Delta z\| = |HV_i - HV_j| / \|z_i - z_j\|$, and reporting the median value over all kNN edges. The VIB model exhibits a lower typical local slope (38.2 HV per latent unit), whereas the non-VIB model shows a higher and more variable slope (97.4 HV per latent unit), consistent with sharper local property variations across nearby latent points when the bottleneck is deterministic (Supplementary Figure 19). Taken together, the increased largest-component connectivity and reduced local slope under VIB provide quantitative support that VIB regularization yields a more connected embedding and a smoother local property landscape, which is directly relevant for latent traversal and gradient-based refinement in the inverse-design procedure, where updates follow gradients in z and therefore benefit from continuity and reduced risk of discontinuous transitions across low-density regions.

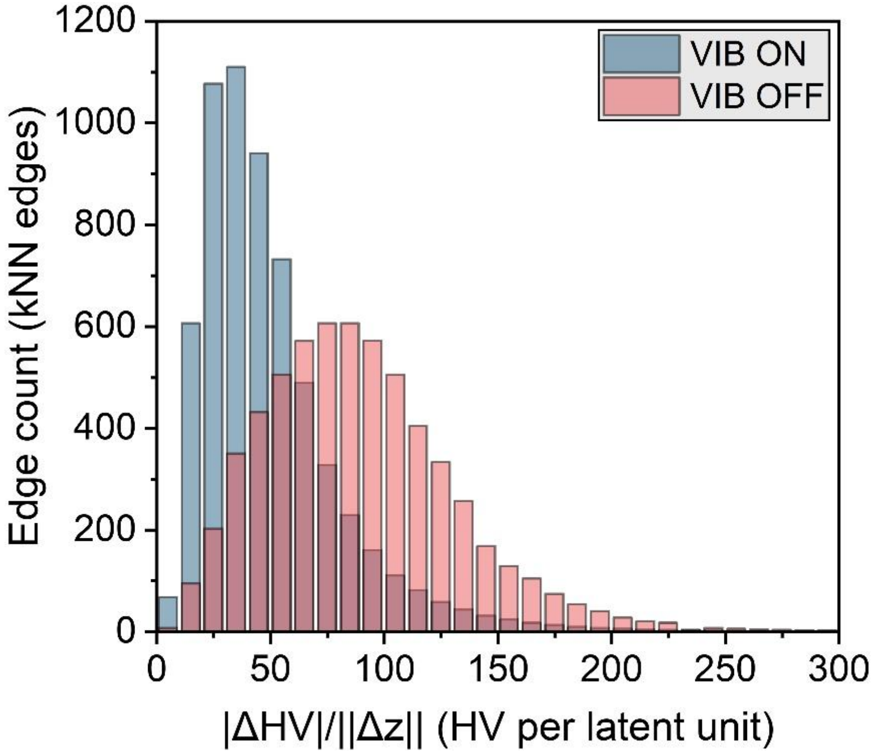

Supplementary Figure 19 – Histogram of the Lipschitz-proxy slope distribution $|\Delta\mathrm{HV}|/\|\Delta \mathrm{z}\|$ computed along k-nearest-neighbor (kNN) edges in the learned latent space for the attention-based model trained with the variational information bottleneck (VIB ON) and the otherwise identical ablation without the VIB term (VIB OFF; deterministic bottleneck). For each of the N = 673 datapoints, the k = 10 nearest neighbors were identified in the latent space using Euclidean distance, and directed edges were formed from each point to its neighbors (total N×k = 6730 kNN edges per model). For each edge (i→j), the local slope was calculated as $|\mathrm{HV_i} - \mathrm{HV_j}|/\|\mathrm{z_i} - \mathrm{z_j}\|$, where HV denotes hardness and z denotes the latent coordinate (units: HV per latent unit). The VIB ON distribution is shifted to lower slopes and is narrower, indicating smoother local variation of hardness with latent displacement, whereas VIB OFF exhibits higher typical slopes and a broader, heavier-tailed distribution, consistent with sharper local property changes and reduced navigability under continuous latent traversal. Bin width is constant across both histograms (as plotted); both panels use identical kNN construction and edge-count normalization. Source data are provided as a Source Data file.

Supplementary Note 7. Inverse Design Gradient-based Optimization

To assess the convergence behavior and effectiveness of our inverse design module, we tracked the optimization loss as the model iteratively modified the latent representation to generate alloy candidates with target hardness values.

Supplementary Figure 20 shows the loss convergence curve for a representative inverse design instance, wherein the latent vector z was initialized from a standard normal distribution and optimized using gradient descent to generate a composition-load pair corresponding to a desired hardness value (e.g., HV = 2500). The optimization objective was to minimize the discrepancy between the predicted hardness from the decoder $f(z)$ and the desired target HV^*, using the following loss function:

$$L_{inv}(z) = (f(z) - HV^*)^2$$

This is a mean squared error (MSE) loss, optimized over the latent variable z, with the decoder weights fixed. The optimization was carried out for 10000 iterations using the Adam optimizer with a learning rate of 1×10^{-4}, and the convergence threshold was defined by the plateauing of the loss below a tolerance of 1×10^{-3}.

The smooth, monotonic decline of the loss function in Supplementary Figure 20 indicates stable convergence of the inverse mapping. In ~7000 iterations, the predicted hardness approaches the target within a small margin, and further optimization yields diminishing returns.

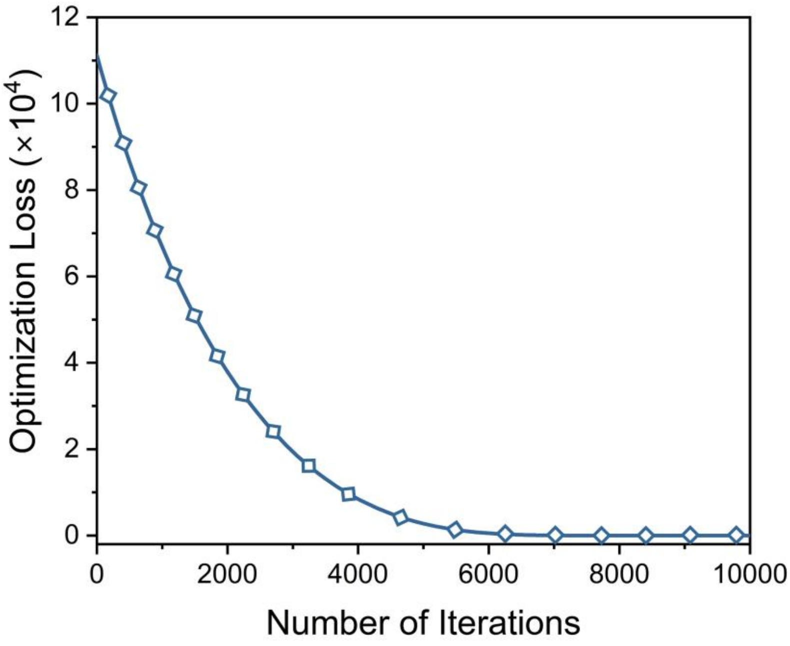

Supplementary Figure 20 – Optimization loss as a function of epochs during the training process for inverse alloy design using gradient-based optimization. Source data are provided as a Source Data file.

Algorithm S3. Inverse design pipeline

Inputs:

- Trained model final_model from Algorithm S1
- Training compositions X_comp_train_fit
- Sample weights w_train_fit; scaler_load_final
- Output normalization (μ_y,σ_y)
- Design load x_{load} (= 0.5 – 10.0 N)
- MC-dropout toggling function set_mc_dropout_inv;
- Inverse-design and optimization hyperparameters.

Outputs:

- Latent samples and diagnostics (mcmc_latent_diagnostics_cheap.csv)
- Ranked candidate table (inverse_design_candidates_FINAL.csv
- Optimized candidates (optimized_candidates_with_uncertainty.csv).

Procedure:

1. **Latent prior construction and "near-peak @ design_load" calibration**
   - A. Fix the design load x_{load} = DESIGN_LOAD_VALUE (0.5-10.0 N) and compute $x_{load,scaled} \leftarrow$ scaler_load(x_{load}).
   - B. Construct an encoder reusing the trained VIB layer
     - A. Define encoder_model with

       $z \leftarrow$ final_model.get_layer("vib_layer").output.
     - B. Encode all training compositions at the fixed design load:

       $Z_{train} \leftarrow$ encoder_model($[X_{comp}^{train}, x_{load,scaled}]$).
   - C. Fit a weighted GMM prior in latent space
     - a. Fit $p_{gmm}(z)$ with K components to Z_{train} using weights w_train_fit.
     - b. Define realism score $\log p_{gmm}(z)$ using gmm.score_samples.
     - c. Set the realism gate logp_thr as a weighted quantile of training $\log \mathrm{p}$.
   - D. Calibrate near-peak behavior (training-based)
     - a. Run BN-safe MC-dropout on a subset of training data at 0.5-10.0 N to compute μ_{train}^{1N}, σ_{train}^{1N}.
     - b. Define training LCB:

       $$LCB_{train}^{1N} = \mu_{train}^{1N} - Z_{conf}\sigma_{train}^{1N}.$$
     - c. Define near-peak mean threshold μ_{peak} as a high quantile of μ_{train}^{1N} and define LCB_TARGET as a conservative high quantile of $\mathrm{LCB}_{train}^{1N}$ restricted to the near-peak set.
     - d. Store SIGMA_REF_Q85 as the 85th percentile of σ_{train}^{1N}.

2. **Mixture-aware multi-chain MCMC in latent space**
   - A. Rebuild decoder_model by wiring the trained layers from final_model:

     x_{comp}←Comp_Dec_*→Comp_Recon.
   - B. No re-training is performed: weights are shared with final_model.
   - C. Define BN-safe MC evaluation for a latent point z (cheap in-MCMC)

a. Decode $\hat{x}_{comp}$ = decoder_model(z), project to simplex (nonnegativity + normalization).

b. Evaluate final_model([$\hat{x}_{comp}$, $x_{load,scaled}$]) under MC-dropout with training=False (BatchNorm frozen) to estimate $\mu(z)$, $\sigma(z)$, and LCB(z).

D. Define the MCMC log-target

a. Hard gate: reject if $\log p_{gmm}(z) < \log p\_thr$.

b. Soft penalties: LCB shortfall below LCB_TARGET, uncertainty above SIGMA_REF_Q85, and latent non-centrality terms.

E. Run mixture-aware multi-chain MCMC

a. Initialize multiple chains from diverse GMM components.

b. Propose z' via a mixture of:

- local Gaussian random walk; and
- global proposals sampled from the fitted GMM.

c. Accept/reject using the Metropolis-Hastings ratio with the implemented proposal correction log_q.

d. After burn-in and thinning, collect pooled latent samples:

$$Z_{samp} = \{z_j\}_{j=1}^{M}.$$

3. Inverse-design candidate evaluation and selection

A. Decode latent samples to candidate compositions

$$X_{cand} \leftarrow \text{decoder\_model}(Z_{samp}),$$

followed by simplex projection.

B. Define composition novelty threshold from training spacing

a. Subsample training compositions $X_{train,sub}$.

b. Compute a reference distribution of pairwise distances within the subsample.

c. Set novel_thr to a high quantile of that reference distribution.

C. Evaluate candidates at 0.5-10 N with high MC-dropout

a. For each candidate, compute S MC draws under BN-safe MC-dropout.

b. Convert scaled predictions back to hardness: HV = $y_{scaled}\sigma_y + \mu_y$.

c. Compute μ_{cand}, σ_{cand}, quantiles, and LCB_{cand}.

D. Acceptance and ranking

Accept if:

- $\log p_{gmm}(z) > \log p\_thr$,
- $\sigma_{HV} \leq 2.5 \times$ SIGMA_REF_Q85,
- novelty distance ≥ novel_thr.

Ranking score (soft_score):

- LCB = μ − z_conf_seed σ
- sigma_pen = max(0, σ – SIGMA_REF_Q85)
- soft_score = LCB + 250*novelty + 0.03*logp − 0.20*sigma_pen^2

4. Gradient-based refinement in latent space

A. Seed selection: Select a small set of high-quality latent seeds from Z_{samp} using feasibility (logp_thr) and near-peak constraint (LCB_TARGET) plus a ranking score.

B. Optimize latent vectors
    a. Treat latent vectors as continuous variables and decode compositions via the same decoder_model.
    b. Evaluate deterministic mean (dropout OFF) and MC uncertainty (dropout ON) at 0.5-10 N.
    c. Optimize an LCB-based objective plus extrapolation guards and regularizers (band penalty, prior penalty, seed-pull, uncertainty cap) exactly as in the code.

C. Re-evaluate optimized candidates with MC-dropout, apply acceptance rules, and save optimized alloy candidates.

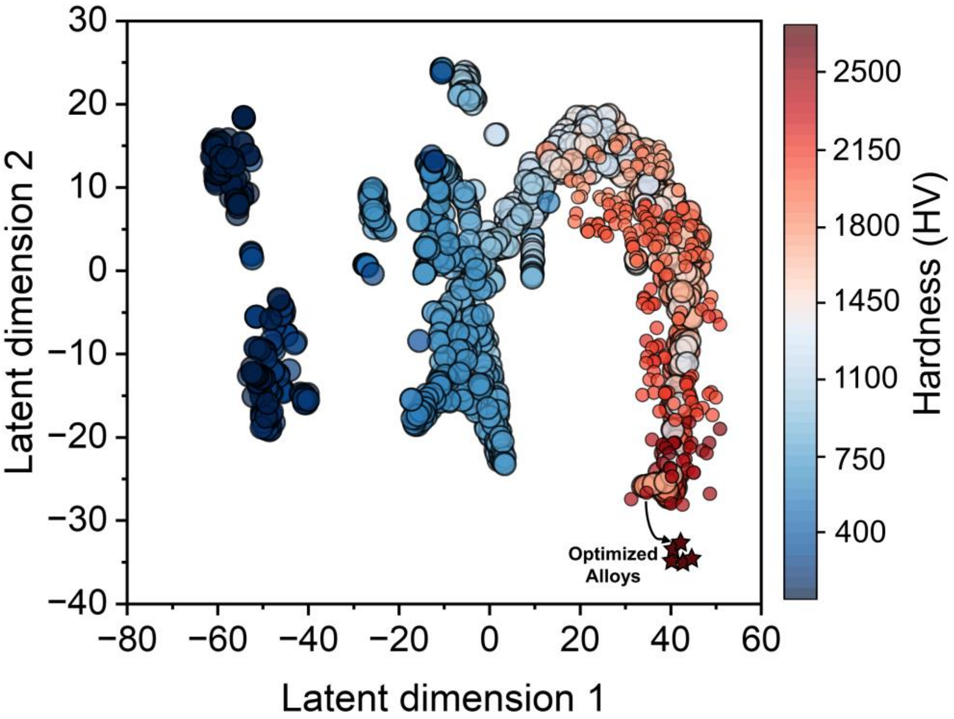

Supplementary Figure 21 – t-SNE projection of the VIBANN latent space showing the original dataset alloys, sampled alloys, and inverse-designed optimized alloys (stars). The color map indicates the predicted Vickers hardness (HV), ranging from low (blue) to high hardness (red). Source data are provided as a Source Data file.

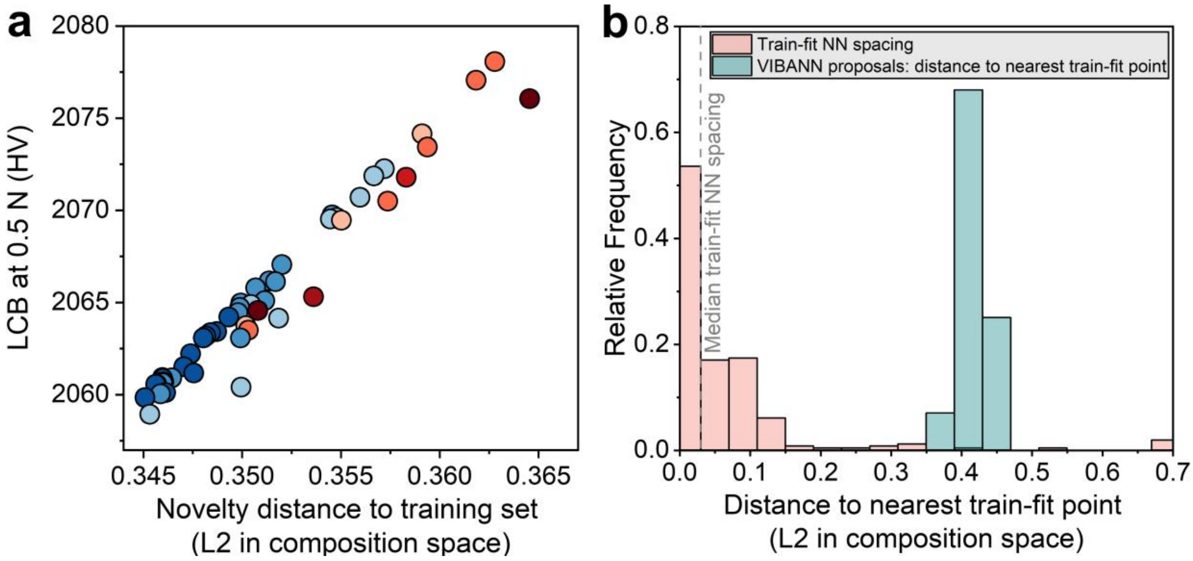

Supplementary Figure 22 – Novelty-performance trade-off and composition space separation of VIBANN inverse design proposals. (a) Conservative performance of VIBANN proposed alloys, quantified by the lower confidence bound at 0.5 N, plotted against novelty distance to the training set defined as the minimum L2 distance in composition space to the nearest training composition. Points are colored by predictive uncertainty. (b) Distribution of composition space distances to the nearest training composition for VIBANN proposals compared with the intrinsic nearest neighbor spacing within the training fit set. The dashed line marks the median nearest neighbor spacing of the training fit set. This comparison shows that the proposed alloys are compositionally distinct relative to typical within-dataset spacings. Source data are provided as a Source Data file.

Supplementary Note 8. Local GP-BO baseline at fixed load (comparison to VIBANN framework)

The inverse-design framework developed in this work combines a regularized latent representation (VIB), an attention-based predictor, calibrated epistemic uncertainty via Monte Carlo dropout, distribution-aware plausibility constraints (latent-density gating), constrained sampling, and gradient-based refinement. Because Gaussian-process Bayesian optimization (GP-BO) is a widely used baseline for materials design, particularly effective when the search is local around the best observed points, we implemented a standard GP-BO pipeline trained on the same dataset and evaluated under the same fixed-load design condition as used for inverse design in the main study. For the GP-BO baseline, the surrogate model was a Gaussian process regressor trained on the joint input space of composition and load, where compositions were represented on the simplex, and the load was standardized using training-fit statistics. The kernel was chosen in a standard form suitable for smooth but nontrivial response landscapes, $k(x) = C{\cdot}\text{Matérn}(\nu = 2.5, \text{ARD}) + \text{White}$, and the kernel hyperparameters (amplitude, ARD length-scales, and noise level) were selected by Bayesian optimization on a calibration split by minimizing the negative log predictive density (NLPD), thereby prioritizing both accuracy and calibrated uncertainty. Inverse design was then performed at the fixed design load used throughout this work (0.5 N), by optimizing only the composition variables on the simplex using a simplex-safe logit-softmax parameterization. We implemented an explicitly local search for new alloys: the acquisition was optimized within a trust region defined around a set of top-performing "anchor" compositions near the design load. Candidate ranking used a conservative lower confidence bound, LCB $(x) = \mu(x) - z\sigma(x)$ with $z = 1.645$ (one-sided 95%), and a repulsion term was used to prevent repeated near-duplicates among sequential proposals. All reported GP-BO candidates, therefore, satisfy the simplex constraint and the explicit locality constraint and are selected under the same risk-aware, conservative criterion used to compare candidates produced by the proposed framework.

Supplementary Figure 23 summarizes the efficacy and behavior of the GP-BO baseline. Supplementary Figure 23a shows parity between predicted and measured hardness on a held-out test split, with uncertainty displayed as vertical intervals. The GP model exhibits strong agreement across the full hardness range, with $R^2 = 0.916$. The corresponding numerical metrics (reported in the inset) confirm competitive predictive performance. Because risk-aware acquisition relies on meaningful uncertainty estimates, we further evaluated predictive interval coverage. Supplementary Figure 23b reports

empirical coverage of $\mu \pm z\sigma$ intervals across multiple z-levels against the nominal Gaussian reference, demonstrating that the GP uncertainties are broadly consistent with the expected coverage behavior on test data. This calibration check provides a direct justification for using LCB as a principled conservative acquisition for the GP-BO baseline rather than an ad-hoc score. Under this reliable surrogate, the local GP-BO loop behaves as expected: Supplementary Figure 23c shows that the best-achieved conservative bound improves rapidly in the early iterations and then plateaus, consistent with efficient exploitation within a locally data-supported region.

Despite this expected local improvement, the remaining panels in Supplementary Figures 23d-f reveal a limitation central to the present design objective. Supplementary Figure 23d plots candidate novelty (minimum $L2$ distance in composition space to the training set) against conservative performance (LCB) for the top GP-BO candidates. The highest-ranked candidates are predominantly concentrated at small novelty distances, indicating that local GP-BO preferentially generates near-neighbor variants of existing compositions when constrained to remain in the region where the surrogate is most reliable. As candidates move farther from the training set, conservative performance does not improve and is typically accompanied by increased uncertainty. This coupling is made explicit in Supplementary Figure 23e, which shows the mean-uncertainty relationship for top candidates: increasing the predicted mean is associated with increasing predictive uncertainty, indicating that the surrogate is being driven toward less well-supported regions even under local constraints. Finally, Supplementary Figure 23f provides a direct clustering diagnostic: the distribution of distances from GP-BO proposals to their nearest training compositions is comparable to the intrinsic nearest-neighbor spacing within the training-fit set. This implies that the GP-BO loop largely samples within the local neighborhood structure already present in the data, rather than generating compositions that are both distinct and conservatively high-performing.

These observations are consistent with the known behavior of local GP-BO in constrained, high-dimensional simplices, where the surrogate is strongest near existing observations, and the optimization is explicitly restricted to a trust region. For the compositionally high-dimensional alloy design problems, however, the target is not only local conservative improvement but the generation of candidates that satisfy a joint set of requirements: distributional support (plausibility), nontrivial novelty, and explicit epistemic-risk control, while still achieving high predicted hardness. In this regime, the VIBANN framework provides technical capabilities that are not available in standard GP-BO without substantial augmentation: the VIB-regularized latent geometry enables stable traversal and refinement in a representation that is learned to be predictive yet

information-controlled; the latent density model provides an explicit on-manifold plausibility constraint; and the uncertainty gate and risk-based acceptance prevent selection of high-mean but weakly supported extrapolations. The GP-BO baseline, therefore, serves as a stringent local comparator: it demonstrates that classical GP-BO can indeed exploit the best-observed region effectively, but it also provides quantitative and diagnostic evidence that such a local loop does not, by itself, meet the "high-performing + nontrivially novel + distribution-supported + risk-controlled" design requirement targeted here.

A quantitative head-to-head comparative summary at the fixed design load is provided in Supplementary Table 5, where both methods are evaluated using the same ranking criterion (LCB with $z = 1.645$). For both pipelines, we report conservative performance of the top candidate (best LCB), central tendency among the best proposals (median LCB of top-5), uncertainty statistics among the leading candidates (median and 90th percentile of σ over top-20), novelty statistics (median and 10th percentile novelty over top-20), and a diversity measure (median minimum pairwise distance among the top-20). In the GP-BO baseline, the best LCB and top-k LCB statistics confirm effective local conservative improvement, but the novelty and diversity statistics remain low, consistent with the clustering behavior diagnosed in Figure 2d-f. In contrast, the VIBANN framework yields candidates that achieve higher conservative performance while simultaneously exhibiting substantially higher novelty and diversity under explicit plausibility and risk constraints.

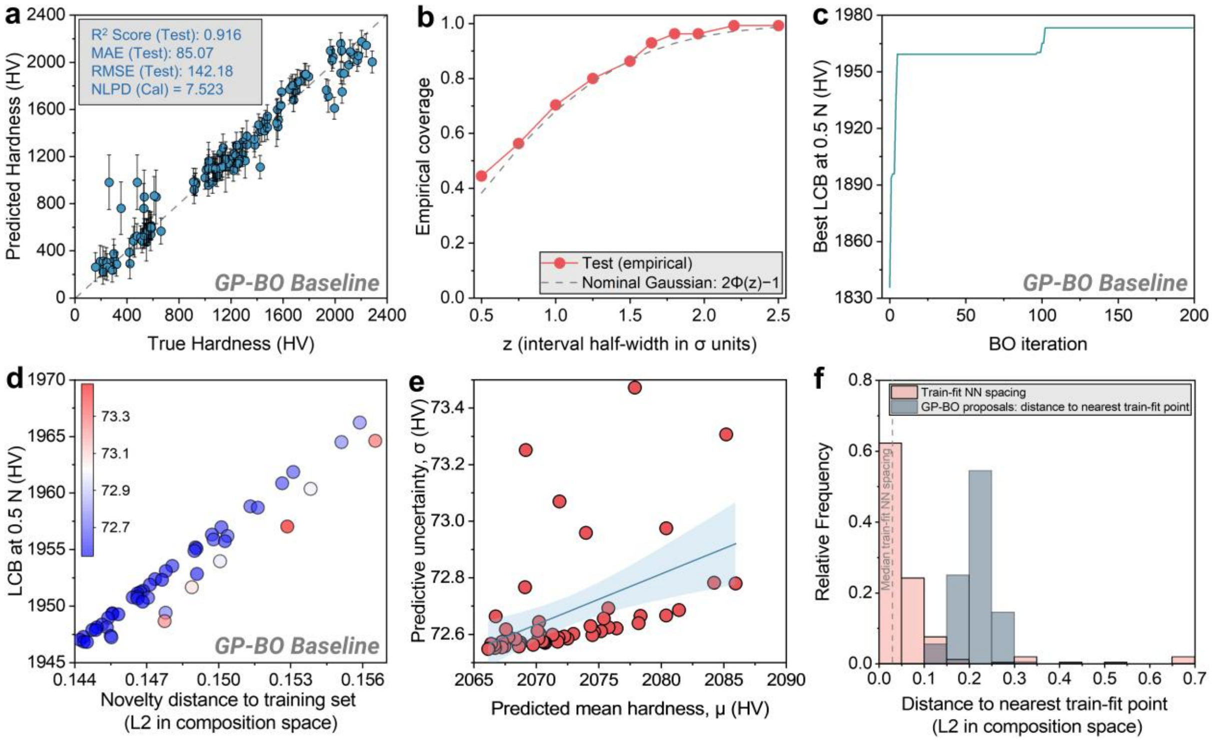

Supplementary Figure 23 – Standard GP-BO baseline on the same composition-load dataset and fixed-load inverse design. (a) Parity plot on the held-out test set (n = 135 observations), showing GP posterior mean predictions against measured hardness. Symbols denote GP posterior mean predictions against measured hardness, and error bars denote ±1σ predictive uncertainty from the GP posterior for each observation. Inset reports test accuracy (R^2, MAE, RMSE) and calibration NLPD. The grey dashed diagonal indicates perfect agreement between predicted and measured values. (b) Empirical coverage of predictive intervals $\mu \pm z\sigma$ compared with the nominal Gaussian reference ($2\phi(z) - 1$) across multiple z-levels, computed from the same n = 135 held-out observations at each z-level, supporting the use of LCB as a risk-aware acquisition. (c) Best-achieved conservative performance (maximum LCB) versus BO iteration for a 200-iteration GP-BO run, demonstrating rapid early gains followed by saturation consistent with local exploitation. (d) Novelty-performance coupling for the top n = 20 GP-BO candidate compositions, where each point is one unique proposed composition evaluated at 0.5 N; showing LCB versus novelty distance (minimum L2 distance in composition space to the training set) with point color indicating predictive uncertainty. (e) Mean–uncertainty trade-off for the same n = 20 candidate compositions, showing predictive standard deviation σ versus predicted mean μ. The blue line denotes the fitted trend, and the shaded band denotes the 95% confidence interval of the trend. (f) Clustering diagnostic comparing the distribution of distances from the same n = 20 GP-BO candidates to their nearest training-fit compositions against the intrinsic nearest-neighbor spacing within the training-fit set (n = 538). Source data are provided as a Source Data file.

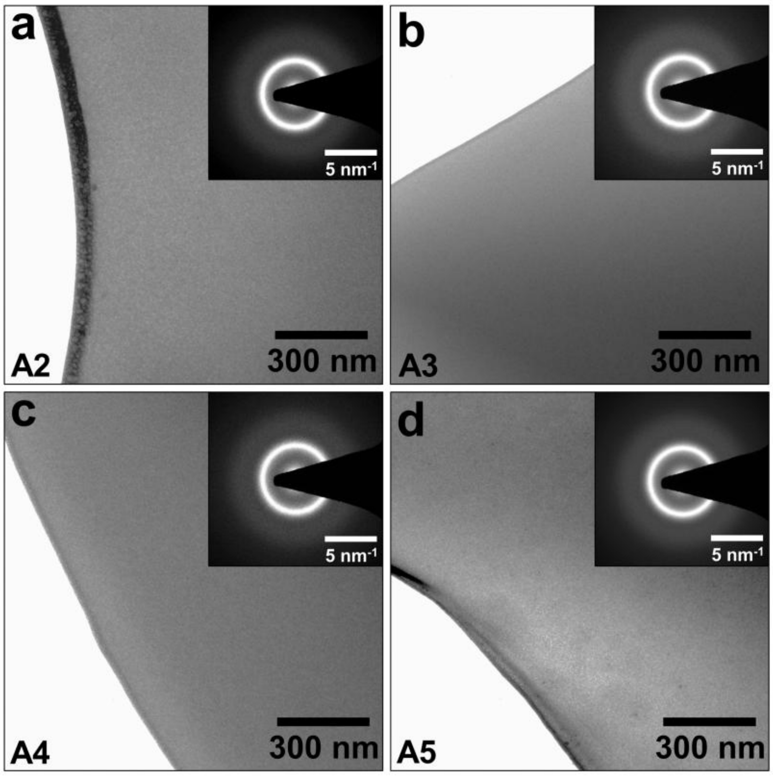

Supplementary Figure 24 – Bright-field TEM micrographs for alloys (a) A2; (b) A3; (c) A4; and (d) A5, reveal featureless contrast typical of amorphous metallic glasses. Insets show selected area electron diffraction (SAED) patterns exhibiting diffuse halos without discrete spots or rings, consistent with a non-crystalline structure.

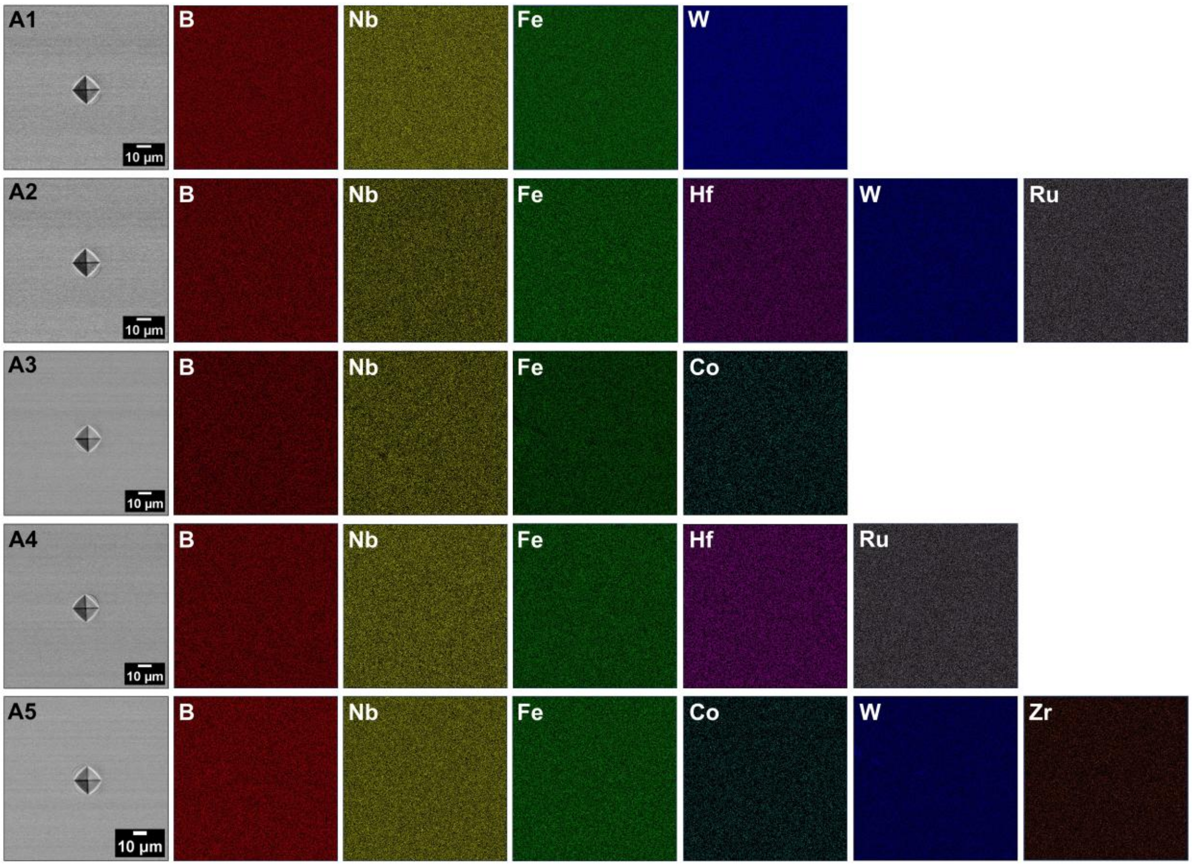

Supplementary Figure 25 – SEM images with corresponding energy-dispersive X-ray spectroscopy (EDS) elemental maps showing the spatial distribution of major constituents in MMG alloys A1 to A5 post-indentation (0.5 N). Each row presents a single alloy with the Vickers indent on the left and associated elemental maps. The uniform contrast and absence of elemental segregation across the indented zone suggest compositional homogeneity and mechanical stability under deformation.

Supplementary Note 9. Atomic Force Microscopy (AFM)-based Pile-Up Correction Methodology

To ensure accurate evaluation of Vickers hardness in high-strength MMGs, we incorporated AFM-based correction to account for pile-up effects that distort the actual contact area. For each alloy and indentation load, AFM topography scans were acquired (Oxford Asylum environmental AFM, tapping mode) over the indent regions. Representative 2D images and depth profiles are shown in Supplementary Figures 26 and 27.

Pile-up was quantified by tracing the height profile along two diagonal indent directions. The actual contact area (A_{AFM}) was determined by integrating the lateral width of the indent and including the outward protrusion from pile-up. The corrected Vickers hardness (HV_{corr}) was then computed using:

$$HV_{corr} = \frac{1.8544 \times F}{A_{AFM}}$$

where F is the applied load. Compared to uncorrected geometrical estimates (assuming ideal pyramidal shape without pile-up), the AFM-corrected values typically showed 6–9% lower hardness under higher loads (>3 N), due to more accurate representation of the plastically deformed contact region. This correction ensured that the experimental hardness values reported throughout the manuscript reflect true material resistance to plastic deformation and not artifacts of surface uplift. The corrected data aligned exceptionally well with the VIBANN-predicted values, further validating the model's fidelity.

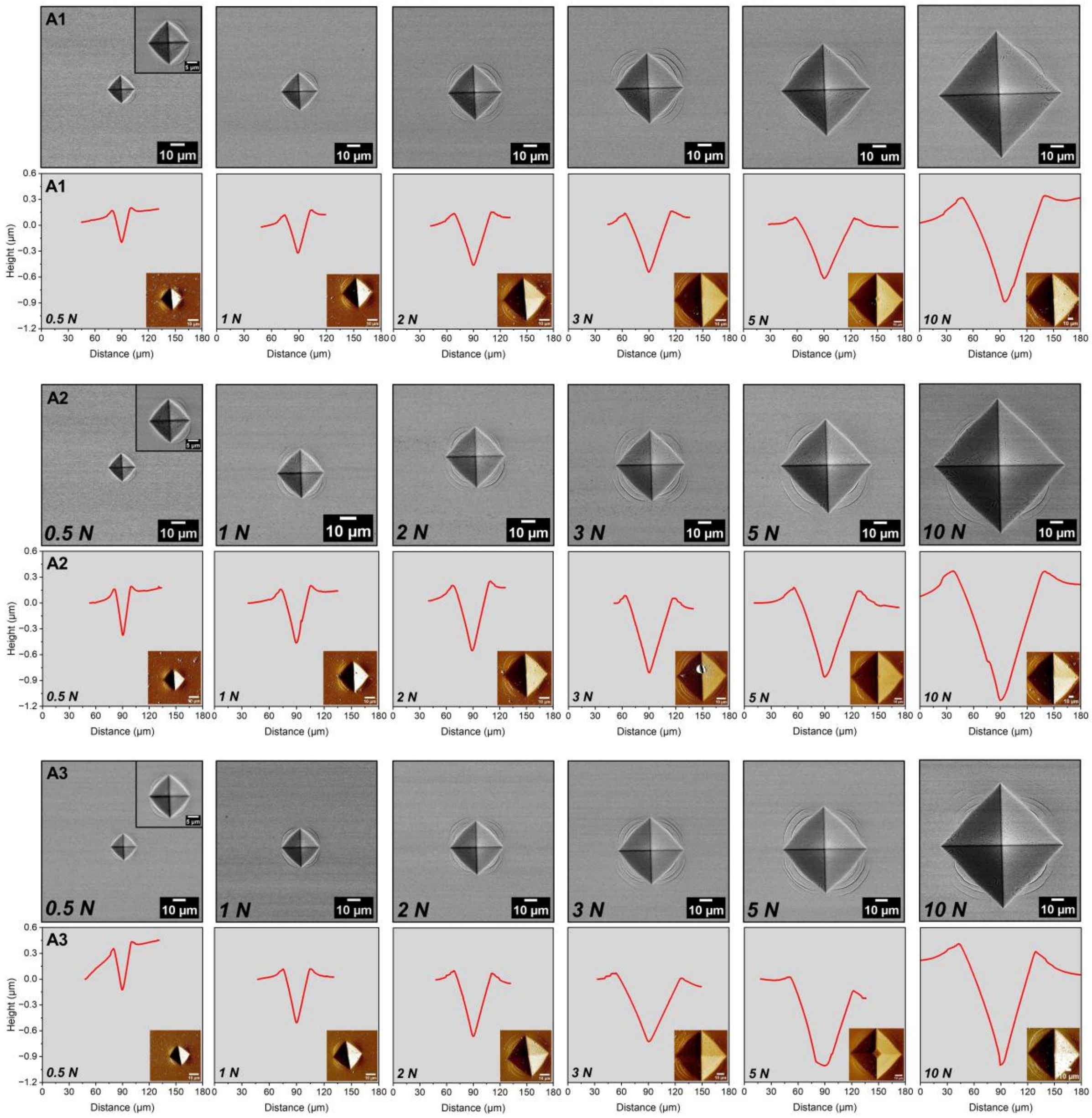

Supplementary Figure 26 – Micrographs and corresponding surface profiles of Vickers indents at varying loads (0.5 N to 10 N) for alloys A1 to A3. For each alloy (A1–A3), top-row images show SEM micrographs of Vickers indentations at different loads, revealing load-dependent deformation behavior and pile-up/sink-in characteristics. The corresponding bottom-row plots show height profiles across the indent diagonals obtained via atomic force microscopy (AFM), highlighting the extent of pile-up with increasing load. Insets in the bottom-row plots present 2D AFM renderings of the indentation morphology. Source data are provided as a Source Data file.

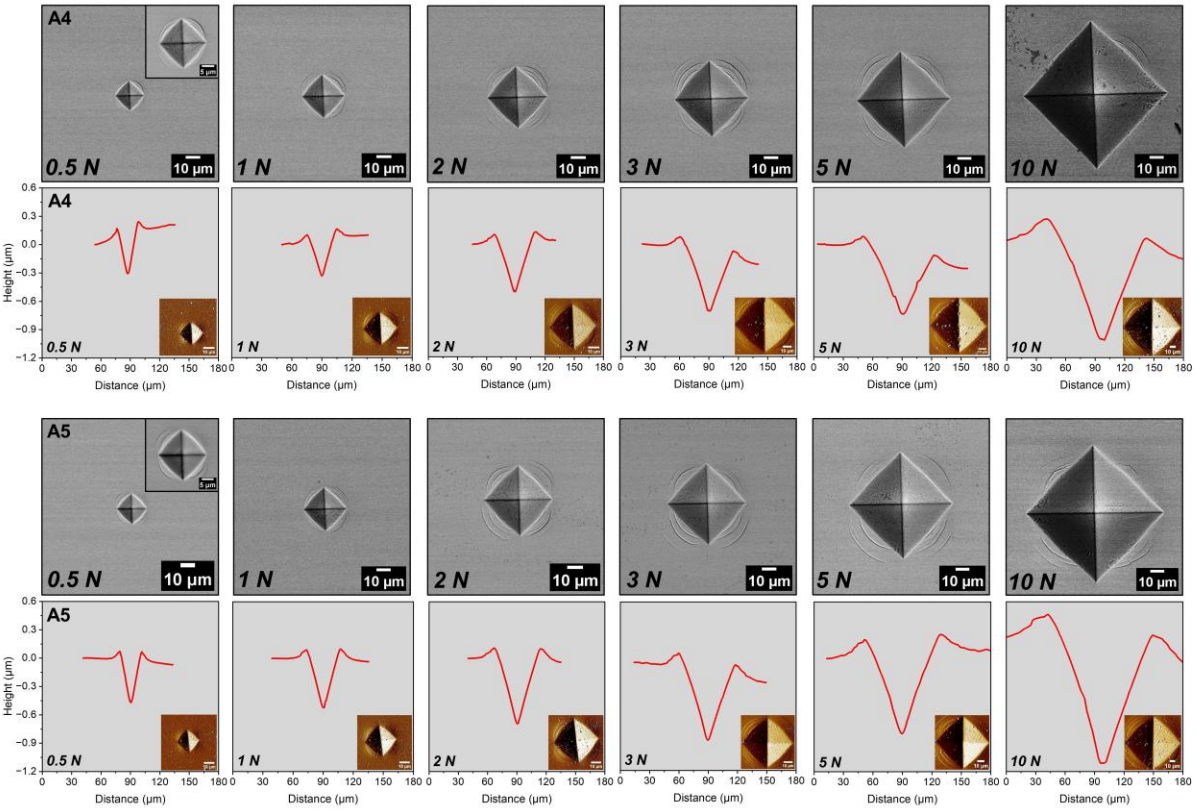

Supplementary Figure 27 – Micrographs and corresponding surface profiles of Vickers indents at varying loads (0.5 N to 10 N) for alloys A4 and A5. The top-row images show SEM micrographs of Vickers indentations at different loads, revealing load-dependent deformation behavior and pile-up/sink-in characteristics. The corresponding bottom-row plots show height profiles across the indent diagonals obtained via atomic force microscopy (AFM), highlighting the extent of pile-up with increasing load. Insets in the bottom-row plots present 2D AFM renderings of the indentation morphology. Source data are provided as a Source Data file.

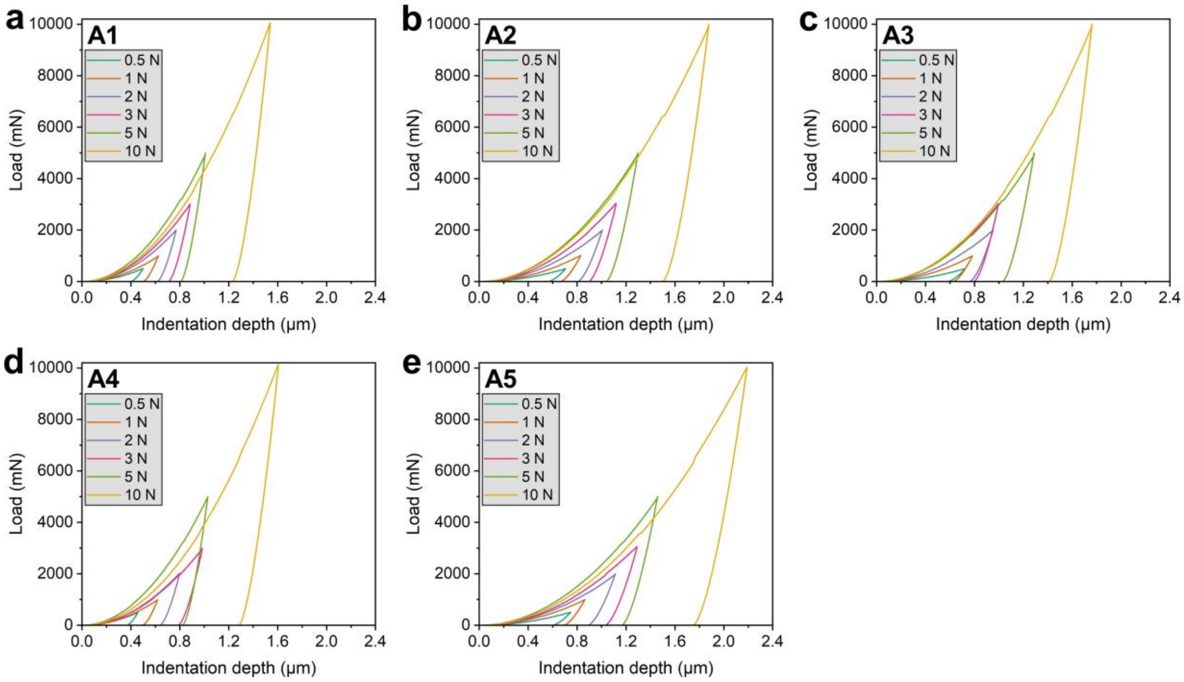

Supplementary Figure 28 – Load-displacement curves obtained from instrumented Vickers indentation on the five inverse-designed bulk MMGs under varying loads. (a-e) Indentation response for alloys A1 through A5, respectively, under applied loads of 0.5-10 N. Source data are provided as a Source Data file.

Supplementary Note 10. Model-based comparison with the nominal B-Nb-Fe ternary baseline

To further examine the role of multicomponent alloying relative to the nominal $B_{72}Nb_{25}Fe_3$ ternary reference from Sarker et al.,[54] we evaluated the trained VIBANN model on this baseline composition and compared its predicted hardness and uncertainty with those of the five experimentally validated inverse-designed alloys at representative bulk indentation loads of 0.5 and 5 N. The corresponding results are summarized in Supplementary Table 8 and Supplementary Figure 29. In the bulk-load regime studied here, the nominal ternary baseline is predicted to have lower hardness than all five selected candidates at both loads. At 0.5 N, the ternary is predicted at 1835.4 HV, whereas A1-A5 BMGs span 1964.9–2342.1 HV. At 5 N, the ternary is predicted at 1571.2 HV, whereas A1-A5 BMGs span 1679.5–2071.7 HV. The corresponding lower-confidence-bound values follow the same ranking, indicating that the improvement is retained under uncertainty-aware evaluation.

To assess whether these gains arise systematically rather than from isolated composition choices, we further traced compositional paths from the nominal ternary baseline toward two representative designed alloys, A1 and A4, while evaluating the model at 0.5 N. As shown in Supplementary Figure 30, gradual alloying from $B_{72}Nb_{25}Fe_3$ to $B_{68}Nb_{24}Fe_4W_4$ results in a monotonic increase in predicted hardness from 1835.4 HV to 2342.1 HV. Likewise, the path from the ternary baseline toward $B_{66}Nb_{21}Fe_4Hf_4Ru_5$ shows a progressive increase from 1835.4 HV to 2070.8 HV. These results indicate that, within the learned bulk-MMG design space, the quaternary and quinary additions do not act as arbitrary perturbations but systematically move the composition toward higher predicted hardness.

These model-based results should be interpreted together with the experimental bulk-synthesis comparison. The nominal $B_{72}Nb_{25}Fe_3$ ternary alloy did not vitrify under the present bulk-casting conditions and instead crystallized (Supplementary Figure 31), whereas the inverse-designed multicomponent alloys formed amorphous bulk rods. Accordingly, the role of the added alloying elements in the present work is not only to tune hardness, but also to shift the system from a crystallization-prone ternary motif toward a bulk-realizable high-hardness compositional region. We also note that previously reported hardness values for related Fe-Nb-B thin-film metallic glasses were obtained at substantially lower indentation loads than those considered here (0.01 N), so caution is warranted when making a direct numerical comparison.

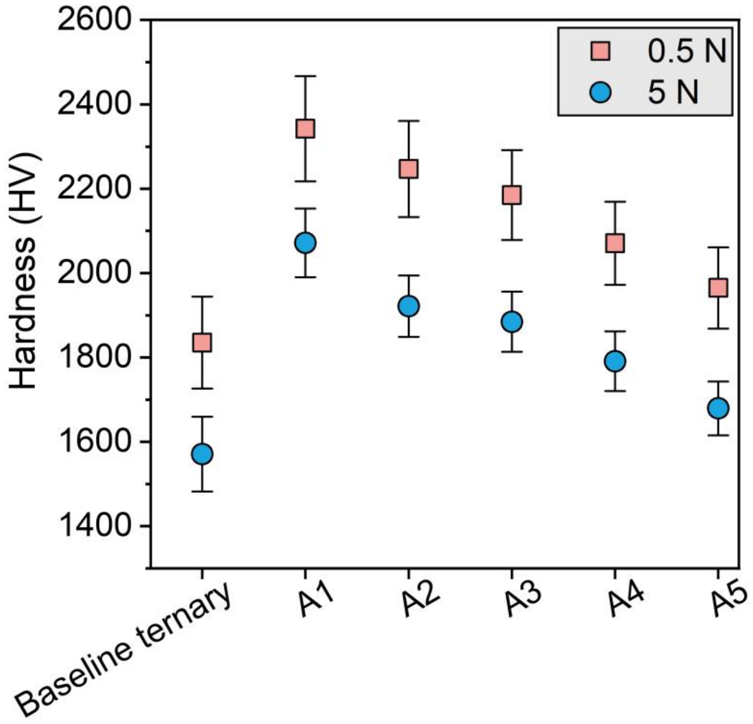

Supplementary Figure 29 – Model-predicted hardness of the nominal $B_{72}Nb_{25}Fe_3$ ternary baseline and the five experimentally validated inverse-designed alloys at indentation loads of 0.5 N and 5 N. Symbols denote the predictive mean from the trained VIBANN model, and error bars denote ±1σ predictive uncertainty from n = 100 stochastic Monte Carlo dropout forward passes for each composition–load point. In the bulk-load regime used for inverse design, all five designed multicomponent alloys are predicted to outperform the ternary baseline. Source data are provided as a Source Data file.

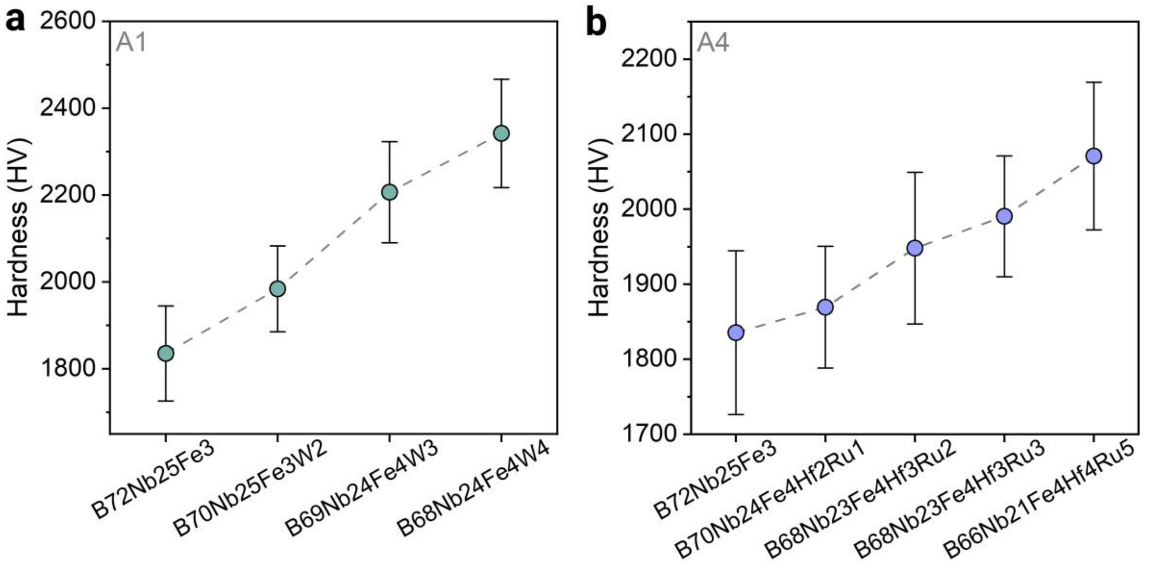

Supplementary Figure 30 – Stepwise model-based compositional evolution from the nominal $B_{72}Nb_{25}Fe_3$ ternary baseline toward representative inverse-designed alloys, evaluated at 0.5 N. (a) Path toward A1, $B_{68}Nb_{24}Fe_4W_4$, through progressive W addition and accompanying adjustment of B, Nb, and Fe; n = 4 compositions are shown, including the baseline ternary and three progressively alloyed compositions. (b) Path toward A4, $B_{66}Nb_{21}Fe_4Hf_4Ru_5$, through progressive Hf/Ru addition and rebalancing of the base ternary chemistry; n = 5 compositions are shown, including the baseline ternary and four progressively alloyed compositions. In both panels, symbols denote predictive mean hardness for each composition evaluated at 0.5 N, and error bars denote ±1σ predictive uncertainty from n = 100 stochastic Monte Carlo dropout forward passes. Grey dashed lines are shown only as guides to the eye. In both cases, the model predicts a monotonic increase in hardness along the compositional path. Source data are provided as a Source Data file.

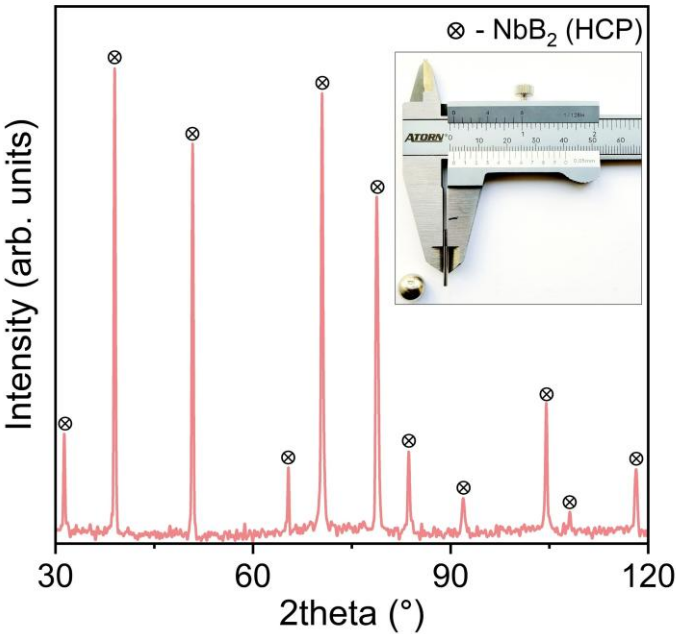

Supplementary Figure 31 – X-ray diffraction pattern of the bulk-cast ternary reference alloy $B_{72}Nb_{25}Fe_3$ prepared using the same arc-melting and suction-casting route used for the inverse-designed alloys. Source data are provided as a Source Data file.

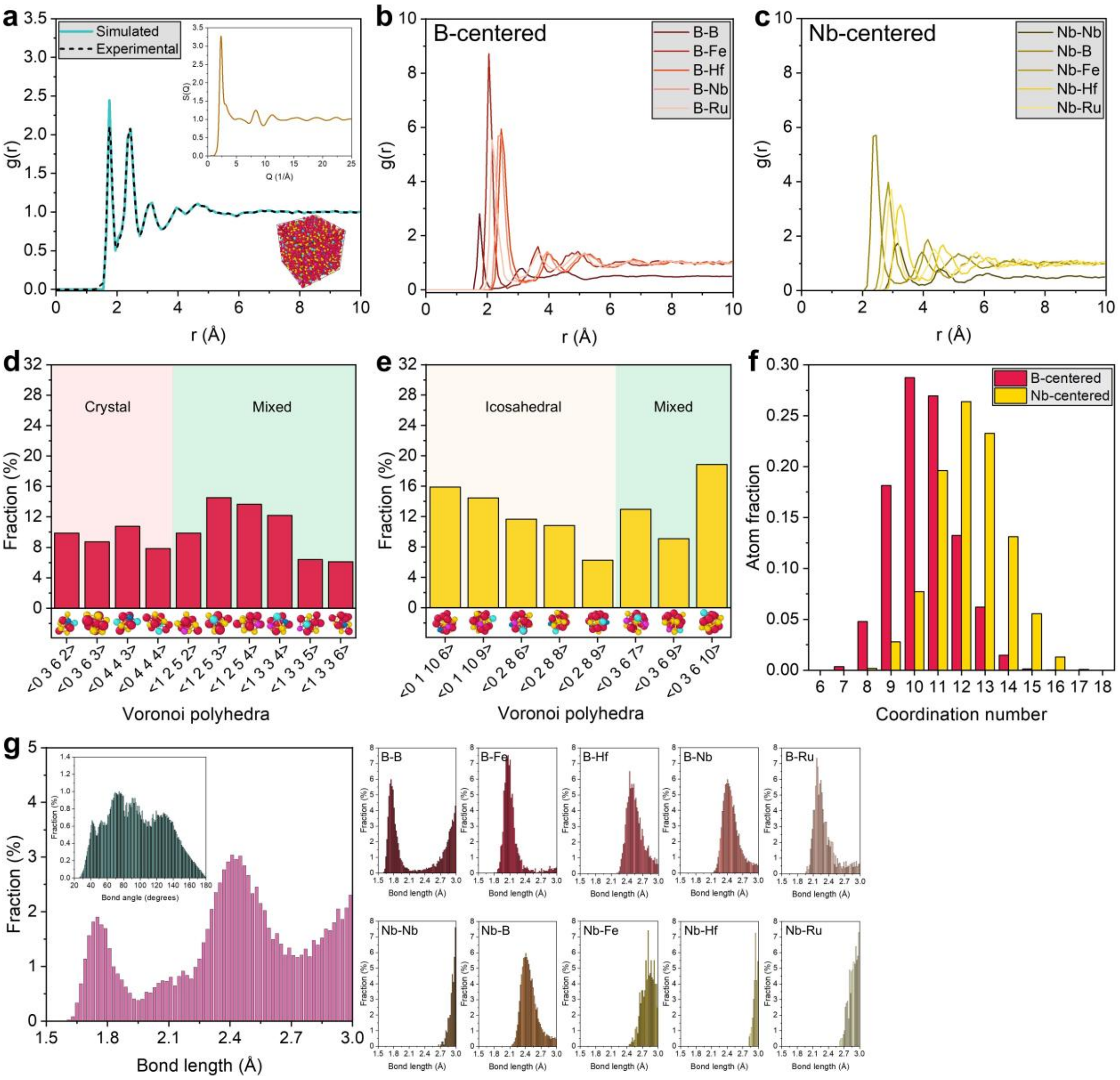

Supplementary Figure 32 – Atomic-scale structural analysis of A2 alloy. (a) Total RDF g(r) for the alloy, showing strong agreement between simulated and experimental data (inset: corresponding structure factor S(Q)), with representative atomic configuration shown. (b, c) Partial RDFs for B-centered and Nb-centered clusters, highlighting prominent nearest-neighbor correlations with constituent elements, and revealing compositional dependence of short-range order. (d) Coordination number histograms for B- and Nb-centered atoms. (e, f) Voronoi polyhedra distributions categorize local environments as crystal-like or icosahedral, with the alloy exhibiting a balanced mixture of both, indicative of medium-range order. (g) Bond length distributions for overall and partial pairs, while the inset shows the overall bond angle distribution. Source data are provided as a Source Data file.

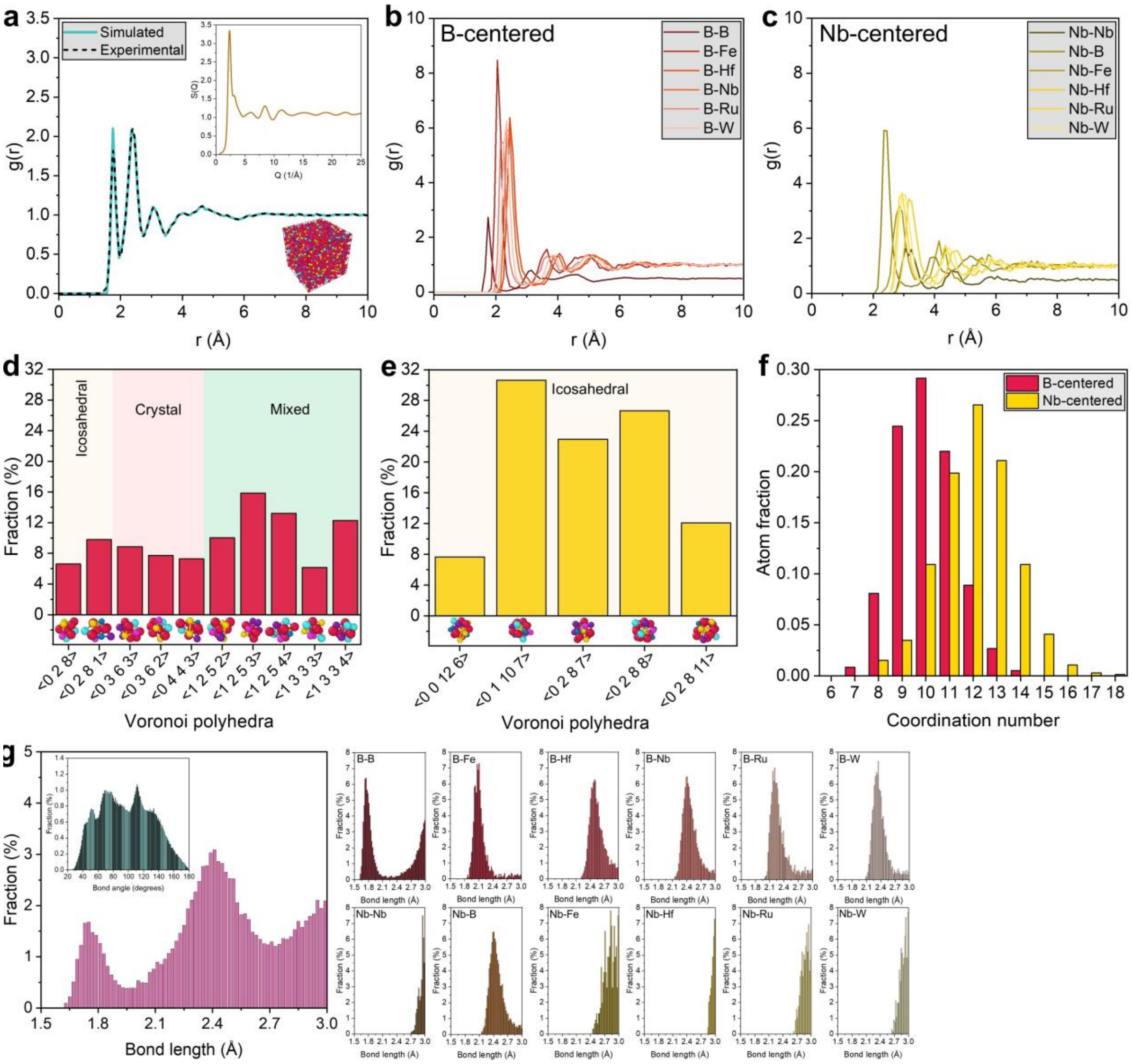

Supplementary Figure 33 – Atomic-scale structural analysis of A3 alloy. (a) Total RDF g(r) for the alloy, showing strong agreement between simulated and experimental data (inset: corresponding structure factor S(Q)), with representative atomic configuration shown. (b, c) Partial RDFs for B-centered and Nb-centered clusters, highlighting prominent nearest-neighbor correlations with constituent elements, and revealing compositional dependence of short-range order. (d) Coordination number histograms for B- and Nb-centered atoms. (e, f) Voronoi polyhedra distributions categorize local environments as crystal-like or icosahedral, with the alloy exhibiting a balanced mixture of both, indicative of medium-range order. (g) Bond length distributions for overall and partial pairs, while the inset shows the overall bond angle distribution. Source data are provided as a Source Data file.

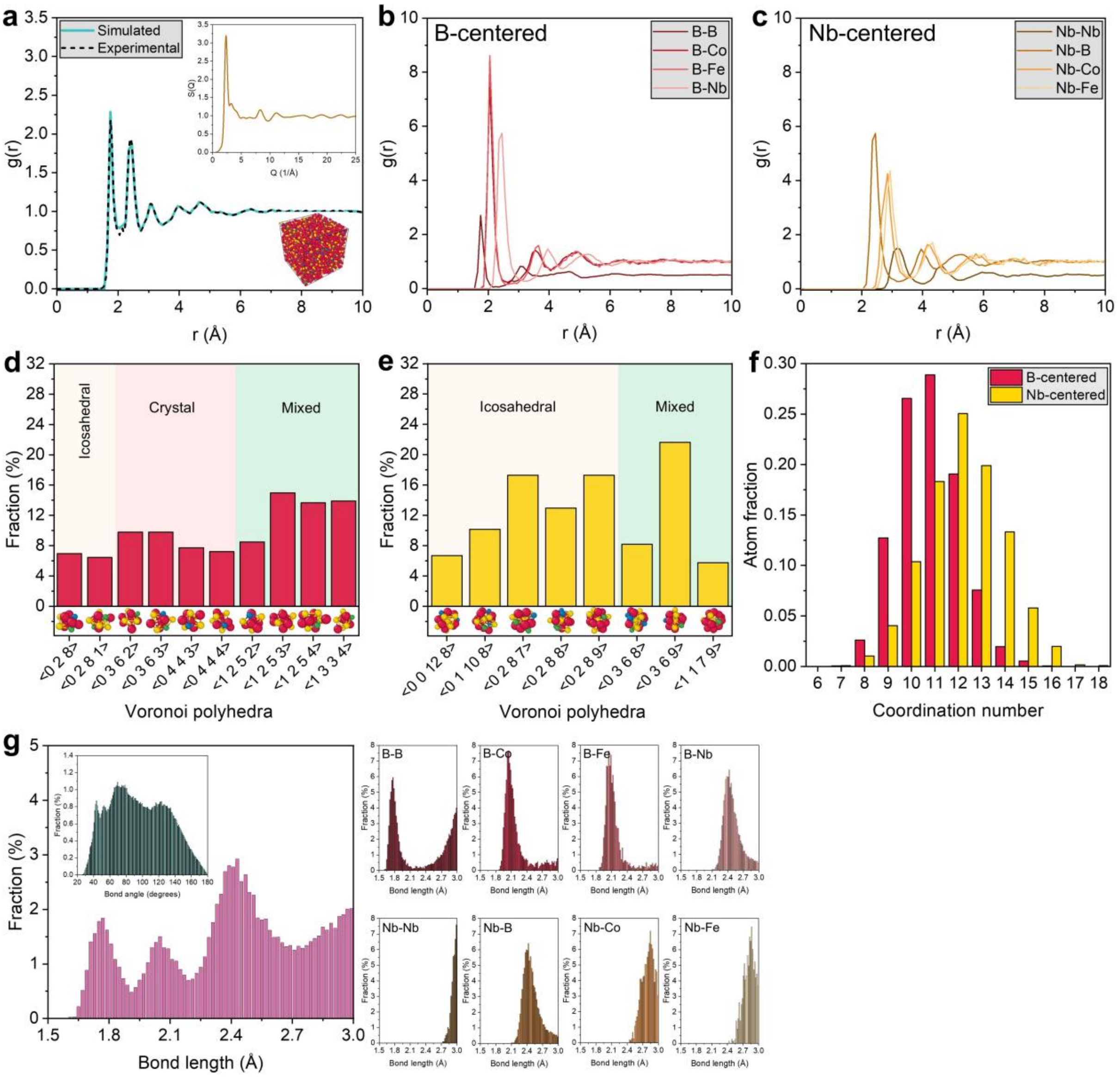

Supplementary Figure 34 – Atomic-scale structural analysis of A4 alloy. (a) Total RDF g(r) for the alloy, showing strong agreement between simulated and experimental data (inset: corresponding structure factor S(Q)), with representative atomic configuration shown. (b, c) Partial RDFs for B-centered and Nb-centered clusters, highlighting prominent nearest-neighbor correlations with constituent elements, and revealing compositional dependence of short-range order. (d) Coordination number histograms for B- and Nb-centered atoms. (e, f) Voronoi polyhedra distributions categorize local environments as crystal-like or icosahedral, with the alloy exhibiting a balanced mixture of both, indicative of medium-range order. (g) Bond length distributions for overall and partial pairs, while the inset shows the overall bond angle distribution. Source data are provided as a Source Data file.

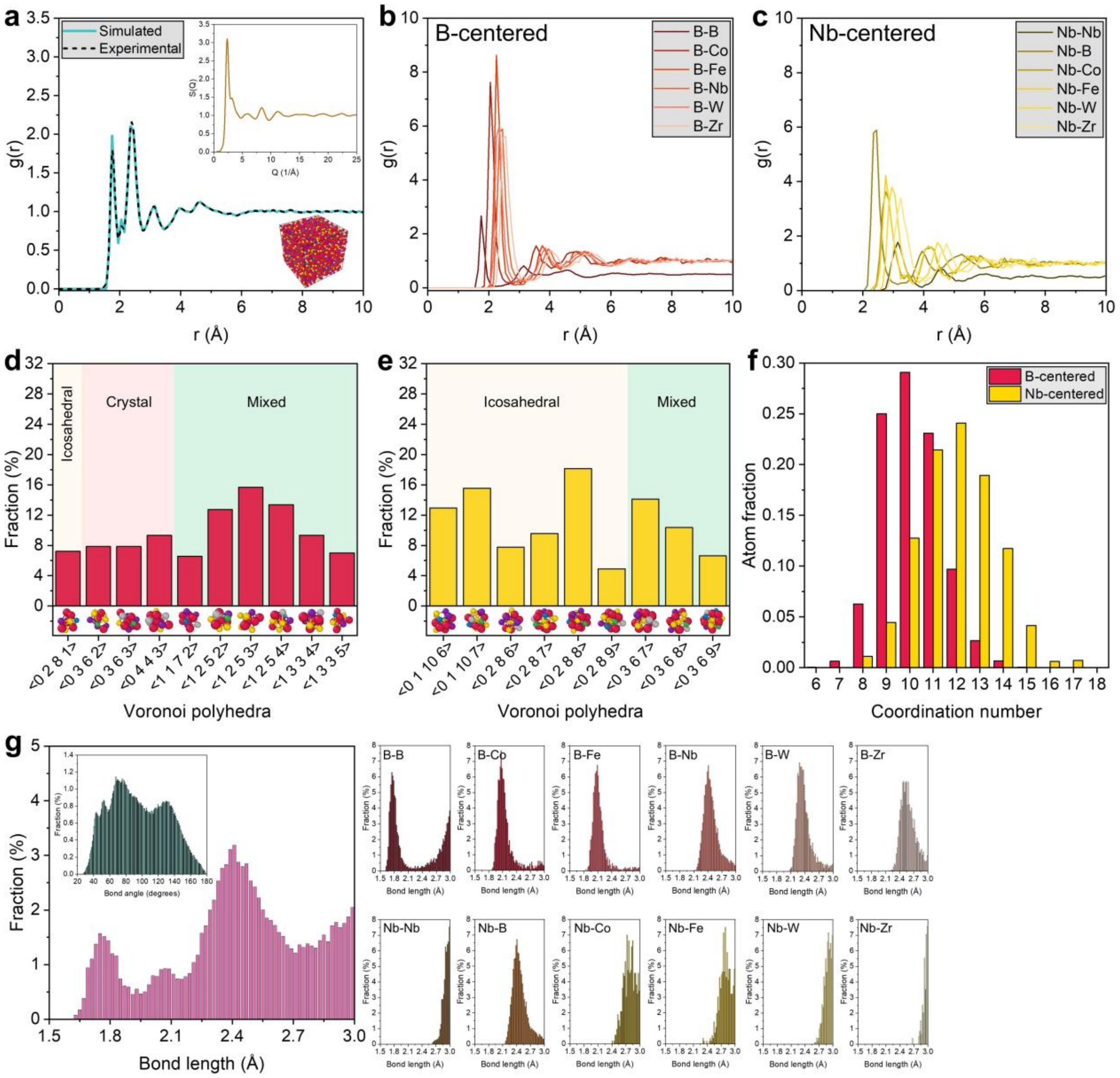

Supplementary Figure 35 – Atomic-scale structural analysis of A5 alloy. (a) Total RDF g(r) for the alloy, showing strong agreement between simulated and experimental data (inset: corresponding structure factor S(Q)), with representative atomic configuration shown. (b, c) Partial RDFs for B-centered and Nb-centered clusters, highlighting prominent nearest-neighbor correlations with constituent elements, and revealing compositional dependence of short-range order. (d) Coordination number histograms for B- and Nb-centered atoms. (e, f) Voronoi polyhedra distributions categorize local environments as crystal-like or icosahedral, with the alloy exhibiting a balanced mixture of both, indicative of medium-range order. (g) Bond length distributions for overall and partial pairs, while the inset shows the overall bond angle distribution. Source data are provided as a Source Data file.

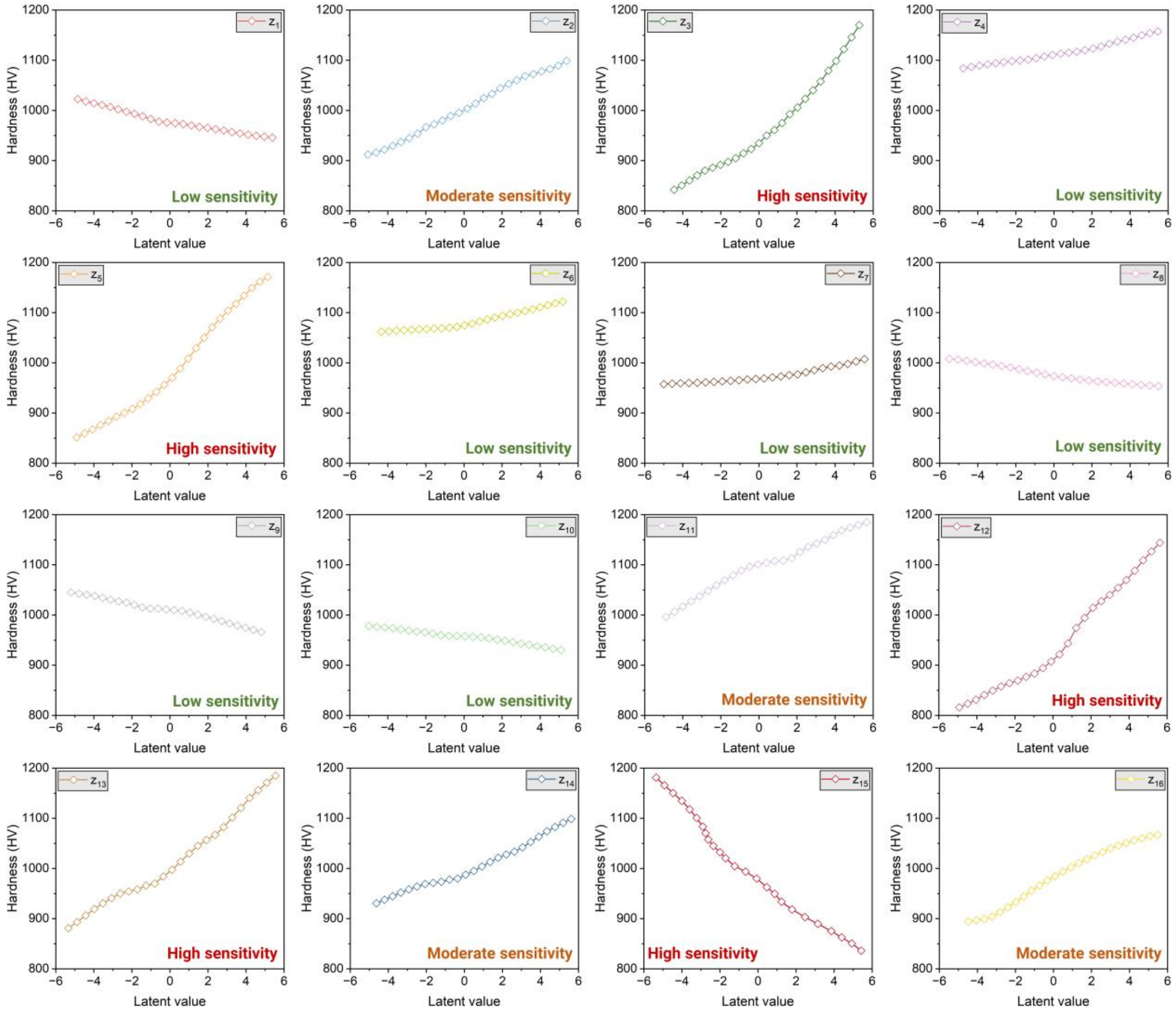

Supplementary Figure 36 – Latent traversal responses from a representative mid-hardness reference point. Traversals were performed from a latent vector sampled near the center of the learned latent distribution, representative of the mid-hardness regime. Each panel perturbs one latent coordinate over the same range, holding the remaining coordinates fixed, and evaluates the corresponding Vickers hardness predicted by the trained VIBANN surrogate. The responses are grouped by sensitivity to highlight that only a limited subset of coordinates produces strong, systematic hardness changes, whereas most yield weak or near-flat responses in this region. Source data are provided as a Source Data file.

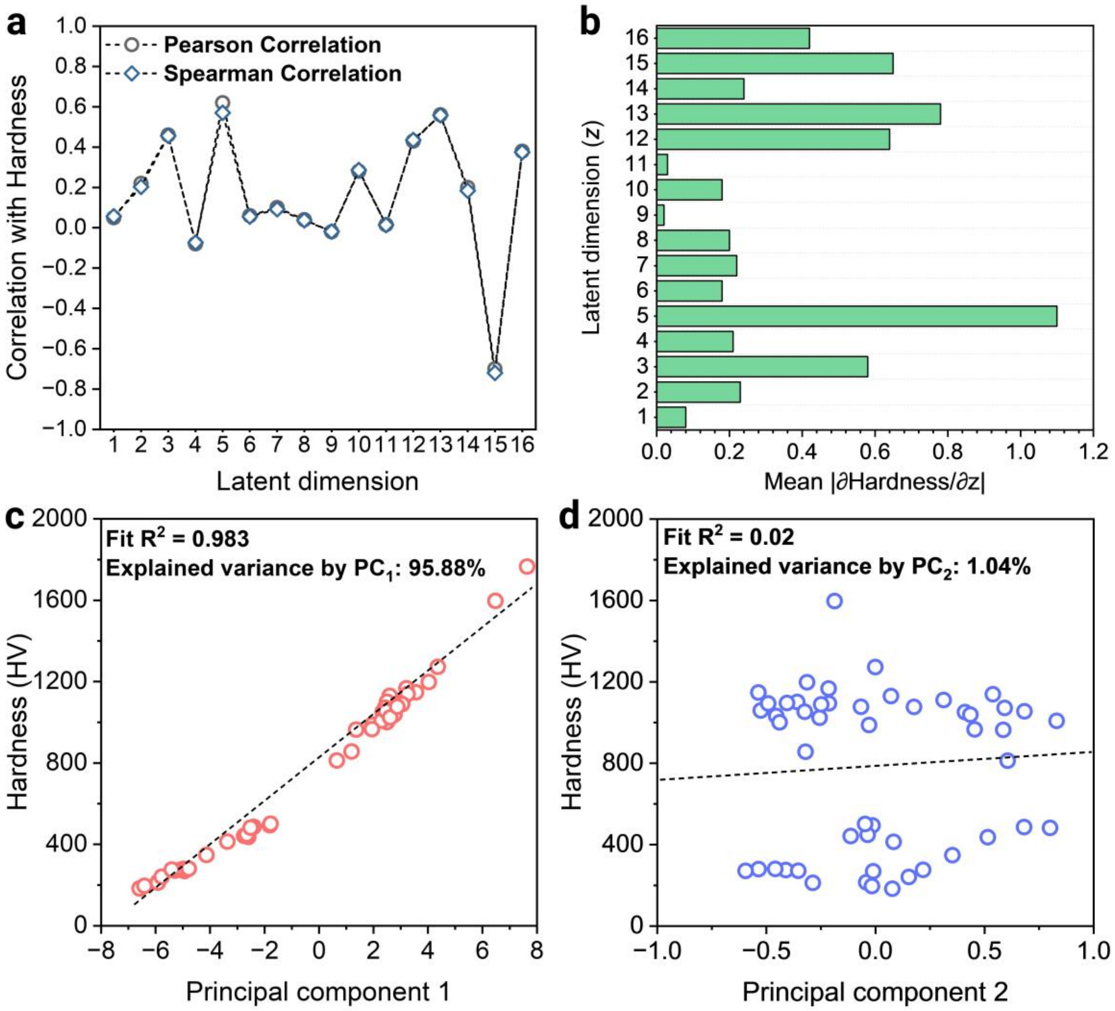

Supplementary Figure 37 – **(a)** Pearson and Spearman correlation coefficients between individual latent dimensions and predicted hardness across the latent space. The strong positive or negative correlations observed across specific dimensions highlight the emergence of meaningful latent variables governing mechanical response, whereas near-zero correlations suggest auxiliary or redundant encoding. (b) Mean absolute gradients ($|\partial \text{Hardness}/\partial z_i|$) are shown for each latent dimension, indicating the relative influence of each dimension on hardness prediction. A few dominant latent directions, particularly dimensions z_3, z_5, z_{12}, z_{13} and z_{15}, exhibit strong control over hardness, while others contribute minimally, suggesting an intrinsically sparse representation of mechanical behavior in the learned latent space. (c)-(d) Principal component analysis (PCA) of the latent space and its relationship to predicted hardness. (c) A strong linear dependence ($R^2 = 0.983$) is observed between the first principal component (PC_1) and predicted hardness, indicating that PC_1 captures the dominant direction of variation in the latent space governing mechanical behavior. (d) In contrast, the second principal component (PC_2) shows negligible correlation with hardness ($R^2 = 0.02$), confirming that hardness variations are predominantly aligned along a single low-dimensional manifold in the latent space. Source data are provided as a Source Data file.

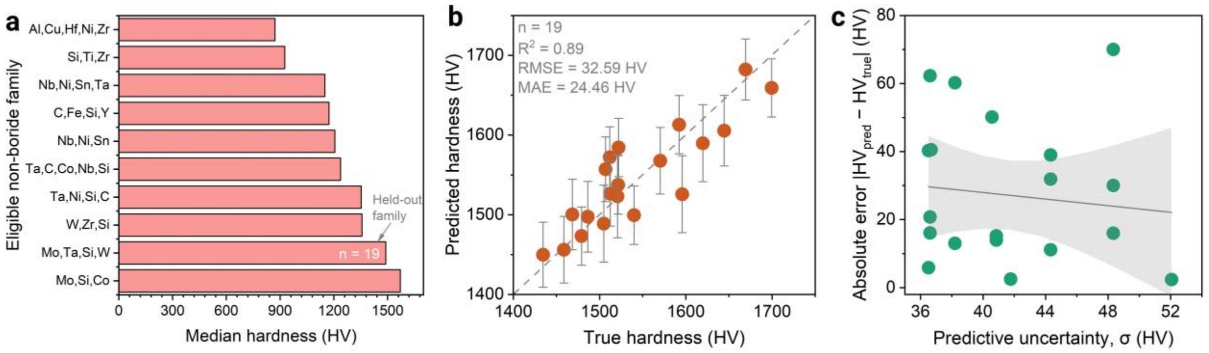

Supplementary Figure 38 – Leave-family-out validation on Mo,Ta,Si,W non-boride metallic-glass family. (a) Eligible non-boride composition families ranked by median hardness. The exact family sizes are n = 4 (Al–Cu–Hf–Ni–Zr), 7 (Si–Ti–Zr), 11 (Nb–Ni–Sn–Ta), 3 (C–Fe–Si–Y), 8 (Nb–Ni–Sn), 12 (Ta–C–Co–Nb–Si), 9 (Ta–Ni–Si–C), 3 (W–Zr–Si), 19 (Mo–Ta–Si–W), and 23 (Mo–Si–Co). The family Mo–Ta–Si–W, containing 19 samples, was chosen for the leave-family-out test based on its comparatively high hardness within the non-boride subset and sufficient representation for meaningful evaluation. (b) True versus predicted hardness for the held-out Mo–Ta–Si–W family after retraining VIBANN with this entire family excluded from the training set. Each point is one composition–load–hardness observation from the held-out family (n = 19). Error bars denote ±1σ predictive uncertainty from n = 100 stochastic Monte Carlo dropout forward passes for each held-out observation; the dashed diagonal indicates perfect agreement. (c) Absolute prediction error as a function of predictive uncertainty for the same n = 19 held-out observations. The grey solid line denotes the linear fit, and the shaded band denotes the 95% confidence interval of the fit. Source data are provided as a Source Data file.

Supplementary Note 11. Integrated Gradients vs. SHAP

We employed Integrated Gradients (IG) to interpret the contribution of elemental features to the predicted hardness in our VIBANN model. IG is a gradient-based method designed for deep neural networks that assigns importance by computing path-integrated gradients from a baseline input (e.g., the mean composition) to the actual input. This produces attributions that reflect the extent to which each input feature contributes to the final prediction, while accounting for complex, non-linear interactions within the model.

Importantly, we did not use SHAP (Shapley Additive Explanations), contrary to what may have been assumed in the review. SHAP operates on the principle of feature permutation and approximates contributions using additive surrogate models under the assumption of feature independence. However, in the context of material compositions, especially metallic glasses, this assumption is fundamentally violated: elemental fractions must sum to 1 (or 100%), leading to strong multicollinearity.

Using SHAP under such constraints can yield misleading attributions, as it may artificially inflate or obscure the contribution of correlated features. On the other hand, Integrated Gradients naturally accommodates correlated inputs, as it relies on the model's internal gradients rather than external permutations. This makes IG a more robust choice for attribution in compositional datasets where feature dependence is intrinsic and unavoidable.

In our implementation:

- We computed IG with respect to a mean composition baseline, ensuring that attributions reflect meaningful deviations from a chemically neutral reference.
- The compositional attributions shown (Figure 6d) therefore represent directional, path-aware contributions to predicted hardness.
- The IG approach also complements our VIBANN architecture, where the deep network and attention layers encode complex feature interactions, making gradient-based interpretations more aligned with the model's internal logic.

Thus, the use of IG provides a chemically faithful, collinearity-aware, and model-specific explanation framework that aligns with the statistical and physical structure of our dataset, justifying its adoption over traditional SHAP-based methods in this work.

Supplementary Table 1 – Search space for all relevant hyperparameters and the best-performing hyperparameters for the various ML models (the best hyperparameters are chosen based using Bayesian optimization and 5-fold cross-validation).

Model	Hyperparameter	Hyperparameters Grid	Best Hyperparameters
Lasso Regression (LR)	Alpha	0.00001-1.0	**0.01**
	Selection	cyclic, random	**cyclic**
Ridge Regression (RR)	Alpha	0.00001-1.0	**0.01**
	Solver	auto, svd, cholesky, lsqr, sparse_cg	**svd**
k-Nearest Neighbours (kNN)	Number of Neighbours	2, 30	**8**
	Weight	uniform, distance	**distance**
	Metric	euclidean, manhattan	**manhattan**
Random Forest (RF)	Number of estimators	10-500	**119**
	Criterion	gini, entropy	**entropy**
	Maximum depth	2-30	**4**
	Minimum sample split	1-20	**7**
	Minimum sample leaf	1-20	**9**
Gradient Boosting (GB)	Number of estimators	10-200	**88**
	Learning rate	0.00001-1.0	**0.001**
	Maximum depth	1-20	**14**
	Minimum sample split	2-20	**3**
Multilayer Perceptron (MLP)	Hidden layers	1-4	**3**
	Hiddent neurons	16-128	**56**
	Learning rate	0.0001-0.01	**0.001**
	Activation function	relu, tanh	**relu**
	Optimizer	adam, sgd	**adam**
	Batch size	16-64	**32**

Supplementary Table 2 – Bayesian optimization trials (each evaluated on 500 epochs) for VIBANN model hyperparameters. Each row represents a trial with different combinations of latent space dimensionality and dropout rate. The green-colored values represent the optimal set of hyperparameters.

Trial	Latent dimensions	Dropout rate	Validation loss
1	16	0.162407	78209.66
2	64	0.432977	81288.48
3	16	0.344741	75980.91
**4**	**16**	**0.336966**	**66856.33**
5	32	0.221846	82268.43
6	64	0.203512	90969.8
7	32	0.4758	84065.19
8	64	0.255471	76982.7
9	64	0.12982	83532.48
10	16	0.408508	82250.8
11	8	0.32354	76237.52
12	16	0.336331	74126.7
13	16	0.375174	91478.5
14	16	0.30182	83728.95
15	8	0.266336	86384.69
16	16	0.375956	77940.33
17	16	0.457565	77747.8
18	16	0.35662	69874.02
19	8	0.40129	81573.59
20	32	0.280562	104393
21	16	0.366795	83708.27
22	16	0.324636	78925.25
23	16	0.311204	71694.66
24	16	0.233734	98093.92
25	16	0.297575	75701.29
26	16	0.414502	67421.09
27	16	0.425836	67377.76
28	8	0.496731	81512.36
29	32	0.431115	99260.94
30	16	0.405499	97054.08
31	16	0.450604	95377.71
32	16	0.353319	103147.3
33	16	0.388022	75024.26
34	16	0.425839	85528.52
35	16	0.34714	74947.69
36	64	0.455167	72850.48
37	16	0.419621	80046.36
38	32	0.493326	81496.05
39	64	0.389084	94550.52
40	16	0.353932	73784.41
41	16	0.162035	69332.17
42	16	0.106008	84718.61

43	16	0.181387	67331.43
44	16	0.175159	72436.13
45	16	0.168923	82405.16
46	64	0.186301	85351.41
47	8	0.142326	82452.47
48	16	0.212331	85479.34
49	32	0.142434	94429.37
50	16	0.240746	78797.32

Supplementary Table 3 – Signed-error statistics for the full test set and upper-hardness subsets.

	N	Mean signed error = mean(predicted − true)	MAE = mean(|predicted − true|)	Fraction within $\pm 1\sigma$ = fraction of points where |predicted − true| ≤ sigma
Full test set	135	-4.79	55.61	0.62
Top 20% true hardness	27	-82.13	122.55	0.41
Top 10% true hardness	14	-122.14	151.8	0.34

Supplementary Note 12. Comparative benchmarking of VIBANN against prior frameworks

Our VIBANN distinguishes itself by integrating capabilities that prior works typically address in isolation. As seen in Supplementary Table 4, earlier models often trade off interpretability for accuracy or lack an inverse design component or uncertainty estimates. For example, Ren et al. achieved autonomous learning with experiments but used relatively opaque random forests,[55] while Jiang et al. built interpretability into a design model but focused on a specific alloy system without a generative mechanism.[56] VIBANN bridges these gaps in the following ways:

1. **Interpretability:** VIBANN embeds interpretability within the model via attention weights highlighting which elemental features drive hardness, and by learning a low-dimensional latent space that correlates with known physical factors. This is a step beyond post-hoc SHAP analysis in works like Pan et al.[57] VIBANN leverages attribution analysis for added rigor, but its self-explanatory latent variables provide intuitive "directions" for improving properties (e.g., it can smoothly vary one latent factor to observe hardness response).
2. **Uncertainty Quantification:** Most earlier studies did not quantify prediction uncertainty (notable exceptions being active learning frameworks from Rao et al.,[58] which used model uncertainty to pick experiments). VIBANN uses Monte Carlo dropout to output confidence intervals on hardness predictions. This uncertainty quantification is crucial in design: it flags less reliable extrapolations, guiding experimentalists to trust VIBANN's high-confidence suggestions – an approach rarely seen in prior alloy design papers.
3. **Attention Mechanisms:** Attention is a core part of VIBANN's architecture, enabling it to capture complex high-order interactions in multicomponent alloys. Prior deep models for alloys seldom employed attention (one recent exception is the NLP-driven corrosion model, which used an LSTM-based text encoder from Sasidhar et al.[59]). VIBANN's use of self-attention in the neural network is novel for materials modeling, allowing the model to weigh the contribution of each element or feature contextually. This directly addresses the challenge noted by previous researchers: that models often treated all features equivalently and lacked a way to prioritize key elemental interactions.
4. **Autonomous Inverse Design:** While generative models (e.g., CVAE by Chen et al.[60]) and Bayesian optimizers (Pan et al.[57]) begin to tackle inverse design, VIBANN offers a fully integrated pipeline. Its GMM-based latent sampling and gradient ascent can

continuously generate candidate alloys with optimized hardness, rather than requiring separate optimization algorithms. Combined with its interpretability and uncertainty quantification, VIBANN effectively closes the loop: it not only points to promising new alloys but also explains why they are promising and how confident the model is. This autonomous loop was validated through the experimental synthesis of five alloys that achieved the targeted exceptionally high hardness, something no previous single framework had achieved in the MGs domain.

In summary, VIBANN represents a substantial advance over prior art by unifying the strengths of various ML approaches. By doing so, it sets a new state of the art for data-driven alloy design – fully autonomous, interpretable, and reliable - thereby bridging the gap between complex deep learning models and practical materials discovery.

Supplementary Table 4 – Comparative benchmarking of VIBANN against seven state-of-the-art machine learning frameworks for alloy design. The comparison spans model architecture, material system, design objectives, inverse design capability, interpretability, predictive performance, experimental validation, and key novelties. VIBANN uniquely combines variational information bottlenecks, attention-based interpretability, uncertainty quantification, and experimentally validated inverse design into a unified framework.

Study	Model	System & Property	Inverse Design	Interpretability	Performance	Validation	Key Contribution
Ren et al., Sci. Adv. 2018[55]	Random forest classifiers (ensemble; Weka), plus stacked model for different processing routes	Multicomponent metallic glasses (MGs); predict glass-forming ability (GFA) under different fabrication methods (melt-spinning vs sputtering)	Yes - iterative active learning loop (ML model proposes compositions, refined with experiments)	Feature importance (RF Gini) analyses; identified key features (e.g. mixing enthalpy, size mismatch) corresponding to known glass formation rules	~90% classification accuracy; AUC improved via stacking; trained on ~6k known alloys (including negative examples from high-throughput experiments)	Yes: 17 new alloys tested in 4 systems; 3 new MG-forming ternary systems discovered	First demonstration of ML-guided alloy discovery loop: integrated ML with high-throughput experiments to accelerate MG discovery by >100×, incorporating processing as a feature. Overcame data bias by including 'failed' alloys, yielding a synthesis-sensitive predictor that improved with each iteration.
Jiang et al., Acta Mater. 2024[56]	Gradient-boosted decision trees (XGBoost) with multi-objective optimization	High-end Al alloy (7xxx series); optimize three properties simultaneously: yield strength, fracture toughness, and stress-corrosion cracking resistance	Yes - model used to search composition space for alloys improving all targets (synchronously)	Post-hoc model explainers (SHAP values) to find dominant elemental effects on each property; model itself is an interpretable ML formulation	R^2 ~0.92 for strength, ~0.85 for toughness; trained on O (10^2) alloy data from literature and industry	Yes: Designed a new Al-Zn-Mg-Cu alloy with higher strength/toughness/corrosion resistance; experimentally verified performance	Multi-property interpretable design: First ML model to concurrently improve strength-toughness-corrosion in Al alloys. Identified key alloying patterns and trade-offs, yielding a new alloy that outperforms prior art, all via an interpretable model.
Sohail et al., Nature 2025[61]	Bayesian active learning with a physics-informed surrogate model (feature-engineered)	High-entropy alloy (Fe-Ni-Co-Al-Ta); maximize yield strength while maintaining high ductility (2 GPa σ_y target)	Yes - iterative active learning over 4 rounds, proposing compositions and using domain filters	Domain knowledge integration (feature engineering & search constraints); no explicit post-hoc explainer, but human-guided feature selection (e.g. valence electron concentration filter)	$R^2 \approx 0.95$ on yield strength; model trained on ~50 known compositions initially, refined with each batch of new data	Yes: 8 alloys synthesized in 4 AL iterations; discovered a 1.8 GPa yield, 25% elongation alloy (record strength-ductility combo)	Domain-guided ML: Pioneered combining expert heuristics with ML. The model embedded phase knowledge (favor FCC via e^- concentration) and microstructure targets, enabling discovery of ultra-strong yet ductile HEA beyond prior limits. Achieved leap in performance with minimal experiments (6 of 8 candidates exceeded prior values).

Rao et al., Science 2022[58]	Active learning (Bayesian optimization) integrating DFT + CALPHAD models in loop	High-entropy Invar alloys (Fe-Ni-Co-Cr-based HEAs); minimize thermal expansion coefficient (Invar effect)	Yes - fully closed-loop design: ML predicts, physics models screen, experiments validate, iterate	Some model explainability via physics: DFT revealed phase-specific volume-change mechanisms for low expansion (no ML-specific explainer used)	N/A (custom acquisition function); Only 17 alloys needed to find optimal; initial data extremely sparse (<0.1% of compositional space)	Yes: 17 alloys made & tested over 3 AL iterations; 2 new HEA Invar alloys found with ultra-low thermal expansion (~2×10^{-6} K^{-1})	Autonomous multi-fidelity design: First active-learning discovery of HEAs. Combined ML with first-principles and thermodynamic simulations in a loop, rapidly finding Invar alloys in an infinite composition space with only a handful of experiments. Demonstrated an efficient path to new materials with targeted thermal/magnetic properties.
Sasidhar et al., Sci. Adv. 2023[59]	Natural language processing (NLP) module + deep neural network (LSTM-enhanced)	Corrosion-resistant alloys (steels, Ni-Cr alloys, Al alloys, HEAs); predict pitting corrosion potential	Yes - model used for composition optimization; textual data allows processing-aware inverse design	Attention-based NLP: automated text parsing of alloy processing descriptions into vectors; Feature importance in trained DNN: identified key derived descriptors (configurational entropy, atomic packing efficiency, etc.) influencing corrosion resistance	Achieved far higher accuracy on pitting potential than prior models (error reduced ~30%) by including text data; ~1000 alloys from literature (with associated text) used for training	No new alloy synthesis (demonstrated on existing data; optimized compositions proposed for further testing)	Novel NLP-driven model: Introduced text-mining of literature to incorporate processing/history into ML. Improved prediction of corrosion performance beyond state-of-the-art by using unstructured data. Also developed a feature-transformed DNN that yields interpretability, highlighting fundamental descriptors for alloy corrosion resistance.
Chen et al., npj Comput. Mater. 2024[60]	CVAE (conditional variational autoencoder) + ANN decoder; data balancing via K-means clustering	Eutectic high-entropy alloys (complex multi-phase alloys); generate compositions with desired dual-/tri-phase microstructure (eutectic fractions)	Yes - generative model directly proposes new ECCA compositions (latent sampling + decoding)	Explainable ML framework: incorporated thermodynamic descriptors (e.g. ΔH, T_m) as features to improve interpretability of latent space; analyzed latent vectors and phase clustering (partial interpretability)	- (Generative model; not primarily evaluated by R^2. Achieved robust generation despite imbalanced data by descriptor engineering)	Yes: Validated 3 new quinary and 1 senary ECCA compositions experimentally (microstructures confirmed as predicted).	Generative inverse design: First deep generative model for multi-phase alloys. The CVAE+ANN framework can directly output viable alloy compositions, overcoming data imbalance by physics-informed features. Discovered complex alloys (4-6 elements) with novel eutectic microstructures not found by prior methods, demonstrating ML-driven phase design in vast composition spaces.

Pan et al., npj Comput. Mater. 2025[57]	Ensemble of regression models (tree-based) for properties + Bayesian optimization for composition search (ZACDS system)	Zn-based alloys (Sn-containing Zn casting alloys); maximize ultimate tensile strength (UTS) under constraints (ductility, hardness)	Yes - Bayesian optimizer suggests new alloy compositions meeting multiple property targets	SHAP value analysis for feature effects on predictions; Particle swarm optimization (PSO) used with SHAP to explore composition-property relations	R^2 ~ 0.90 for UTS, elongation, and hardness (each); ~200 alloys in training set	Yes: Designed a new high-strength Zn-Al-Cu-Mg alloy; achieved UTS > 350 MPa with good elongation as predicted	Automated design platform: Developed an end-to-end Zn Alloy Composition Design System (ZACDS) combining ML and optimization. Integrated interpretability (SHAP) to guide design and successfully produced a novel Zn alloy matching prediction. Illustrates a generalizable approach for rapid alloy development under property constraints.
**Our VIBANN framework**	**VIB + Attention Neural Network (β-VAE style latent info bottleneck + self-attention layers); Monte Carlo dropout for uncertainty quantification; Gaussian mixture model for autonomous generation**	**Multicomponent metallic glasses (B-Nb-Fe-W-Co/Hf/Ru/Zr); target Vickers hardness (exceptionally high hardness >2200 HV)**	**Yes - latent-space inverse design (gradient ascent in learned latent dimensions to maximize hardness, then decode to composition)**	**Intrinsic interpretability via attention weights (identify which elements drive hardness) and disentangled latent factors (smoothly relate to properties); + attribution analysis on model predictions for robust feature attribution**	**$R^2 \approx 0.95$ on hardness; uncertainty-calibrated predictions (dropout); ~539 alloys used for training (data from literature)**	**Yes: Five bulk metallic glass alloys predicted and fabricated; all achieved record hardness >2200 HV (up to ~2450 HV), exceeding nearly all known MMGs.**	**VIBANN combines key advancements: interpretability, attention, uncertainty quantification, and automated inverse design, in one framework. It learns physically meaningful latent representations of composition-property relationships, enabling an autonomous design loop that outputs experimentally validated alloys with exceptional performance. It also bridges deep learning and metallurgical knowledge in a fully closed, explainable design cycle.**

Supplementary Table 5 – Quantitative comparison of local GP-BO baseline versus the VIBANN framework at fixed load (0.5 N). Candidates are ranked by conservative performance LCB = $\mu - z\sigma$ with z = 1.645. Reported statistics are computed on the top-k candidates as indicated. Novelty and diversity are computed identically for both frameworks using the same L2 distance in composition space and the same training-fit reference set.*

**Framework**	**Search variable**	**Constraints enforced during search**	**Best LCB (top-1)**	**Median LCB (top-5)**	**Median σ (top-20)**	**90th %ile σ (top-20)**	**Median novelty (top-20)**	**10th %ile novelty (top-20)**	**Diversity (top-20)**
GP-BO	composition (simplex)	simplex + trust region	1966.2	1964.3	72.66	73.01	0.148	0.147	0.0211
VIBANN	latent + decoded simplex	plausibility + novelty + σ-cap + risk + (simplex)	2078.4	2075.2	66.42	67.15	0.358	0.352	0.0782

***Note:** LCB is a conservative score used for ranking under uncertainty. It is not intended to match the mean prediction (reported in the manuscript for newly discovered alloys), and is naturally lower in high-uncertainty regimes.

Supplementary Table 6 – Bulk chemical compositions of the final optimized MMGs measured by energy dispersive spectroscopy.

Alloy ID	Alloy	B (at.%)	Nb (at.%)	Fe (at.%)	Co (at.%)	Hf (at.%)	Ru (at.%)	W (at.%)	Zr (at.%)
A1	$B_{68}Nb_{24}Fe_4W_4$	67.8 ± 5.4	24.2 ± 2.2	4.1 ± 0.4	–	–	–	3.9 ± 0.4	–
A2	$B_{62}Nb_{12}Fe_4Hf_8Ru_6W_8$	61.5 ± 5.0	12.3 ± 1.0	3.9 ± 0.4	–	7.7 ± 0.7	5.9 ± 0.5	7.8 ± 0.6	–
A3	$B_{64}Nb_{23}Fe_5Co_8$	63.7 ± 5.1	23.1 ± 2.1	4.9 ± 0.5	8.3 ± 0.7	–	–	–	–
A4	$B_{66}Nb_{21}Fe_4Hf_4Ru_5$	65.5 ± 5.2	20.7 ± 1.9	3.7 ± 0.4	–	4.1 ± 0.4	5.0 ± 0.4	–	–
A5	$B_{61}Nb_{18}Fe_3Co_5W_8Zr_5$	60.8 ± 5.1	18.0 ± 1.6	3.1 ± 0.3	4.9 ± 0.5	–	–	7.8 ± 0.6	5.4 ± 0.5

Supplementary Table 7 – Structural descriptors derived from molecular dynamics simulations highlighting short-range order and their correlation with hardness in inverse-designed MMGs.

Alloy	Primary peak height (RDF)	Primary peak sharpness (Height/FWHM)	Primary peak area	Primary to second peak height ratio	Distance between peaks	Fraction of atoms with CN=12 and 13	Fraction of atoms with CN=12 and 13 (B-centered clusters	Fraction of atoms with CN=12 and 13 (Nb-centered clusters	Icosahedral cluster fraction	Mean bond length (Å)	Bond length spread	Icosahedral Peak Index
**A1**	2.64	17.99	0.631	1.46	0.614	0.339	0.258	0.494	0.358	2.405	0.375	0.513
**A2**	2.45	15.43	0.68	1.56	0.687	0.3	0.194	0.497	0.321	2.408	0.371	0.081
**A3**	2.02	12.82	0.601	1.24	0.581	0.257	0.116	0.476	0.541	2.415	0.358	0.144
**A4**	2.3	18.67	0.313	1.5	0.747	0.345	0.266	0.433	0.415	2.394	0.372	0.347
**A5**	1.99	14.57	0.429	1.23	0.63	0.266	0.124	0.43	0.413	2.413	0.353	0.079

Supplementary Table 8 – Model-predicted hardness metrics for the nominal ternary baseline from Sarker et al.[54] and the experimentally validated inverse-designed alloys at representative loads.

**Alloy**	**Composition**	**Predicted hardness at 0.5 N**	**Predicted uncertainty at 0.5 N**	**LCB at 0.5 N**	**Predicted hardness at 5 N**	**Predicted uncertainty at 5 N**	**LCB at 5 N**
Baseline ternary	$B_{72}Nb_{25}Fe_3$	1835.4	109.2	1620.8	1571.2	88.6	1397.1
A1	$B_{68}Nb_{24}Fe_4W_4$	2342.1	124.7	2097.6	2071.7	81.3	1912.3
A2	$B_{62}Nb_{12}Fe_4Hf_8Ru_6W_8$	2246.8	114	2023.3	1921.6	72.7	1779.1
A3	$B_{64}Nb_{23}Fe_5Co_8$	2185.2	106.5	1976.4	1884.7	71.3	1744.9
A4	$B_{66}Nb_{21}Fe_4Hf_4Ru_5$	2070.8	98.3	1878.1	1791.2	70.8	1652.4
A5	$B_{61}Nb_{18}Fe_3Co_5W_8Zr_5$	1964.9	96.3	1776.1	1679.5	63.8	1554.4

Supplementary Table 9 – Top boron-free inverse-designed bulk MMG candidates. Predicted hardness (μ), predictive uncertainty (σ), and lower confidence bound (LCB = μ – 1.96σ) are reported together with composition-space novelty (L2 distance from the nearest training composition) and latent-space log-likelihood as a measure of plausibility within the learned design manifold. Candidates are ranked to highlight non-boride, chemically plausible high-hardness directions identified under the same uncertainty-aware inverse-design framework.

Alloy	Predicted hardness (HV)	Uncertainty (HV)	LCB (HV)	Novelty (L2)	Latent space log-likelihood
$Mo_{38}W_8Si_{24}Ta_8Zr_5Co_{12}Y_5$	1876.4	78.34	1722.8	0.23	-7.6
$Mo_{36}Si_{24}Ta_{10}Hf_8Fe_{17}Y_5$	1864.0	84.92	1697.5	0.39	-7.3
$W_{52}Zr_{18}Si_{18}Ta_6Ti_6$	1855.1	75.67	1706.8	0.28	-8.2
$W_{43}Zr_{14}Hf_{13}Si_{22}Ta_8$	1847.6	81.48	1687.9	0.19	-7.8
$Ta_{52}W_{16}Zr_{12}Si_{10}Ti_{10}$	1834.2	86.11	1665.4	0.35	-8.2
$Mo_{32}Ta_{14}Si_{20}Zr_{20}Hf_{14}$	1826.8	79.03	1671.9	0.32	-8.0
$W_{46}Ta_{12}Zr_{18}Hf_8Si_{16}$	1811.2	82.76	1648.6	0.26	-8.2
$W_{42}Ta_{10}Zr_{16}Ti_{10}Si_{14}C_8$	1802.6	74.58	1656.4	0.37	-8.0
$Ta_{48}Ni_{18}Si_{14}C_{10}Hf_{10}$	1784.9	85.37	1617.5	0.24	-8.2
$Ta_{55}Ti_{10}Zr_{14}Hf_9Si_{12}$	1761.4	80.25	1604.1	0.30	-7.6

Supplementary references

1 Zhu, C. L., Wang, Q., Wang, Y. M., Qiang, J. B. & Dong, C. Co–B–Si–Ta bulk metallic glasses designed using cluster line and alloying. Journal of Alloys and Compounds **504**, S34-S37, (2010).

2 Taghvaei, A. H., Stoica, M., Prashanth, K. G. & Eckert, J. Fabrication and characterization of bulk glassy $Co_{40}Fe_{22}Ta_8B_{30}$ alloy with high thermal stability and excellent soft magnetic properties. Acta Materialia **61**, 6609-6621, (2013).

3 Yu, H. B., Yu, P. & Bai, H. Y. Lutetium and thulium based rare earth bulk metallic glasses. Journal of Non-Crystalline Solids **354**, 4539-4542, (2008).

4 Zhai, S. et al. Effects of pre-compression on the microstructure, mechanical properties and corrosion resistance of $Cu_{47.5}Zr_{47.5}Al_5$ bulk metallic glasses. Journal of Non-Crystalline Solids **481**, 383-390, (2018).

5 Tang, J. et al. Effect of atomic mobility on the electrochemical properties of a $Zr_{58}Nb_3Cu_{16}Ni_{13}Al_{10}$ bulk metallic glass. Electrochimica Acta **267**, 222-233, (2018).

6 Choi-Yim, H., Tokarz, M., Bilello, J. C. & Johnson, W. L. Structure and properties of $Ni_{60}(Nb_{100-x}Ta_x)_{34}Sn_6$ bulk metallic glass alloys. Journal of Non-Crystalline Solids **352**, 747-755, (2006).

7 Iqbal, M. et al. Mechanical properties and ion irradiation of bulk amorphous $Zr_{55}Cu_{30}Al_{10}Ni_5$ alloy. Journal of Non-Crystalline Solids **353**, 2452-2458, (2007).

8 Li, S., Wang, R. J., Pan, M. X., Zhao, D. Q. & Wang, W. H. Formation and properties of $RE_{55}Al_{25}Co_{20}$ (RE=Y, Ce, La, Pr, Nd, Gd, Tb, Dy, Ho and Er) bulk metallic glasses. Journal of Non-Crystalline Solids **354**, 1080-1088, (2008).

9 Betancourt, I. & Baez, S. Influence of Ta, Y substitutions on the thermal stability, microhardness and magnetic properties of melt spun Fe–B–Si amorphous alloys. Journal of Non-Crystalline Solids **355**, 1202-1205, (2009).

10 Pan, Y., Cao, H., Ding, L., Zhang, C. & Chang, Y. A. Novel bulkier copper-rich ternary metallic glasses from computational thermodynamics. Journal of Non-Crystalline Solids **356**, 2168-2171, (2010).

11 Shi, L.-l. & Xu, J. Mg based bulk metallic glasses: Glass transition temperature and elastic properties versus toughness. Journal of Non-Crystalline Solids **357**, 2926-2933, (2011).

12 Liu, G. B., Gao, P., Xue, Z., Yang, S. Q. & Zhang, M. L. Study on the formation of new Mg–Cu–Ti–Y quaternary bulk metallic glasses with high mechanical strength. Journal of Non-Crystalline Solids **358**, 3084-3088, (2012).

13 Zhang, L., Pang, S., Chen, C. & Zhang, T. Formation and mechanical properties of La–Al(–Ga)–C bulk metallic glasses with high content of carbon. Journal of Non-Crystalline Solids **403**, 18-22, (2014).

14 Sun, Y. et al. Comparison of mechanical behaviors of several bulk metallic glasses for biomedical application. Journal of Non-Crystalline Solids **406**, 144-150, (2014).

15 Xu, T., Pang, S., Li, H. & Zhang, T. Corrosion resistant Cr-based bulk metallic glasses with high strength and hardness. Journal of Non-Crystalline Solids **410**, 20-25, (2015).

16 Cao, Q. P. et al. Enhanced plasticity in Zr–Cu–Ag–Al–Be bulk metallic glasses. Journal of Non-Crystalline Solids **412**, 35-44, (2015).

17 Zhang, K., Gao, X., Dong, Y., Xing, Q. & Wang, Y. Effect of annealing on the microstructure, microhardness, and corrosion resistance of $Ni_{62}Nb_{33}Zr_5$ metallic glass and its composites. Journal of Non-Crystalline Solids **425**, 46-51, (2015).

18 Li, H., Pang, S., Liu, Y., Liaw, P. K. & Zhang, T. In vitro investigation of Mg–Zn–Ca–Ag bulk metallic glasses for biomedical applications. Journal of Non-Crystalline Solids **427**, 134-138, (2015).

19 Yu, K. M. et al. Glass forming ability and bending plasticity evolutions in Zr-Co-Al bulk metallic glasses and their structural origin. Journal of Non-Crystalline Solids **488**, 52-62, (2018).

20 Hasani, S., Rezaei-Shahreza, P., Seifoddini, A. & Hakimi, M. Enhanced glass forming ability, mechanical, and magnetic properties of $Fe_{41}Co_7Cr_{15}Mo_{14}Y_2C_{15}B_6$ bulk metallic glass with minor addition of Cu. Journal of Non-Crystalline Solids **497**, 40-47, (2018).

21 Hu, F., Luo, Q. & Shen, B. Thermal, magnetic and magnetocaloric properties of FeErNbB metallic glasses with high glass-forming ability. Journal of Non-Crystalline Solids **512**, 184-188, (2019).

22 Zhang, S. et al. Role of Co content on the microstructure and anti-corrosion performance of high-hardness $AlNiYCo_x$ high entropy metallic glasses. Journal of Non-Crystalline Solids **576**, 121268, (2022).

23 Zhang, S. et al. An ab initio simulation and experimental studies of the glass-forming ability and properties of $Al_{86}Ni_{(14-x)}Zr_x$ (x = 1~7) alloys. Journal of Non-Crystalline Solids **586**, 121566, (2022).

24 Huang, X. M., Chang, C. T., Chang, Z. Y., Inoue, A. & Jiang, J. Z. Glass forming ability, mechanical and magnetic properties in Fe–W–Y–B alloys. Materials Science and Engineering: A **527**, 1952-1956, (2010).

25 Nabialek, M., Dospial, M., Szota, M., Olszewski, J. & Walters, S. Manufacturing of the bulk amorphous $Fe_{61}Co_{10}Zr_{2+x}Hf_{3-x}W_2Y_2B_{20}$ alloys (where x=1, 2, 3) their microstructure, magnetic and mechanical properties. Journal of Alloys and Compounds **509**, S155-S160, (2011).

26 Han, Y. et al. New Fe-based soft magnetic amorphous alloys with high saturation magnetization and good corrosion resistance for dust core application. Intermetallics **76**, 18-25, (2016).

27 Li, T. H. et al. Significantly enhanced mechanical properties of ZrAlCo bulk amorphous alloy by microalloying with Ta. Intermetallics **93**, 162-168, (2018).

28 Li, H. et al. Biodegradable Mg–Zn–Ca–Sr bulk metallic glasses with enhanced corrosion performance for biomedical applications. Materials & Design **67**, 9-19, (2015).

29 Zhang, W., Jia, F., Zhang, Q. & Inoue, A. Effects of additional Ag on the thermal stability and glass-forming ability of Cu–Zr binary glassy alloys. Materials Science and Engineering: A **459**, 330-336, (2007).

30 Cheney, J. & Vecchio, K. Development of quaternary Fe-based bulk metallic glasses. Materials Science and Engineering: A **492**, 230-235, (2008).

31 Xu, T. et al. Tuning glass formation and brittle behaviors by similar solvent element substitution in (Mn,Fe)-based bulk metallic glasses. Materials Science and Engineering: A **626**, 16-26, (2015).

32 Wang, J. et al. Hard rhenium–boron–cobalt amorphous alloys with a wide supercooled liquid region. Materials Science and Engineering: A **645**, 122-125, (2015).

33 Jia, F. et al. Effect of Co concentration on thermal stability and magnetic properties of (Fe,Co)–Nb–Gd–B glassy alloys. Journal of Alloys and Compounds **504**, S129-S131, (2010).

34 Jian, H., Luo, W., Tao, S. & Yan, M. Mechanical and magnetic properties of $(Fe_{72}Mo_4B_{24})_{100-x}Tb_x$ (x=4, 5, 6, 7at.%) bulk glassy alloys. Journal of Alloys and Compounds **505**, 315-318, (2010).

35 Li, J. W., He, A. N. & Shen, B. L. Effect of Tb addition on the thermal stability, glass-forming ability and magnetic properties of Fe–B–Si–Nb bulk metallic glass. Journal of Alloys and Compounds **586**, S46-S49, (2014).

36 Liu, G. B., Gao, P., Yang, S. Q., Xue, Z. & Zhang, M. L. Effects of Zn addition on the glass forming ability and mechanical properties of Mg–Cu–Gd bulk metallic glasses. Journal of Alloys and Compounds **588**, 59-63, (2014).

37 Li, J. W. et al. Electronic-structure origin of the glass-forming ability and magnetic properties in Fe–RE–B–Nb bulk metallic glasses. Journal of Alloys and Compounds **617**, 332-336, (2014).

38 Hua, N., Liao, Z., Chen, W., Huang, Y. & Zhang, T. Effects of noble elements on the glass-forming ability, mechanical property, electrochemical behavior and tribocorrosion resistance of Ni- and Cu-free Zr-Al-Co bulk metallic glass. Journal of Alloys and Compounds **725**, 403-414, (2017).

39 Geng, Y. X. et al. Super-high hardness of (Fe,Co)-B-Si-Zr/Hf bulk glassy alloys. Journal of Alloys and Compounds **753**, 351-355, (2018).

40 Huang, D., Li, Y., Yang, Y., Zhu, Z. & Zhang, W. Soft magnetic Co-based Co–Fe–B–Si–P bulk metallic glasses with high saturation magnetic flux density of over 1.2 T. Journal of Alloys and Compounds **843**, 154862, (2020).

41 Li, J. et al. Thermal stability, magnetic and mechanical properties of Fe–Dy–B–Nb bulk metallic glasses with high glass-forming ability. Intermetallics **46**, 85-90, (2014).

42 Liang, X. et al. Roles of Y and Fe contents on glass-forming ability, thermal stability, and magnetic properties of Co-based Co–Fe–Y–B bulk metallic glasses. Intermetallics **132**, 107135, (2021).

43 Shamlaye, K. F., Laws, K. J. & Löffler, J. F. Exceptionally broad bulk metallic glass formation in the Mg–Cu–Yb system. Acta Materialia **128**, 188-196, (2017).

44 Tishby, N., Pereira, F. C. & Bialek, W. The information bottleneck method. arXiv preprint physics/0004057, (2000).

45 Alemi, A. A., Fischer, I., Dillon, J. V. & Murphy, K. Deep variational information bottleneck. arXiv preprint arXiv:1612.00410, (2016).

46 Kingma, D. P. & Welling, M. (Banff, Canada, 2013).

47 Achille, A. & Soatto, S. Information Dropout: Learning Optimal Representations Through Noisy Computation. IEEE Transactions on Pattern Analysis and Machine Intelligence **40**, 2897-2905, (2018).

48 Lawson, D. et al. in 2018 IEEE International Conference on Acoustics, Speech and Signal Processing (ICASSP). 5799-5803.

49 Xie, T. & Grossman, J. C. Crystal Graph Convolutional Neural Networks for an Accurate and Interpretable Prediction of Material Properties. Physical Review Letters **120**, 145301, (2018).

50 Jang, E., Gu, S. & Poole, B. Categorical reparameterization with gumbel-softmax. arXiv preprint arXiv:1611.01144, (2016).

51 Bahdanau, D., Cho, K. & Bengio, Y. Neural machine translation by jointly learning to align and translate. arXiv preprint arXiv:1409.0473, (2014).

52 Vaswani, A. et al. Attention is all you need. Advances in neural information processing systems **30**, (2017).

53 Jain, S. & Wallace, B. C. Attention is not explanation. arXiv preprint arXiv:1902.10186, (2019).

54 Sarker, S. et al. Discovering exceptionally hard and wear-resistant metallic glasses by combining machine-learning with high throughput experimentation. Applied Physics Reviews **9**, 011403, (2022).

55 Ren, F. et al. Accelerated discovery of metallic glasses through iteration of machine learning and high-throughput experiments. Science Advances **4**, eaaq1566, (2018).

56 Jiang, L. et al. Synchronously enhancing the strength, toughness, and stress corrosion resistance of high-end aluminum alloys via interpretable machine learning. Acta Materialia **270**, 119873, (2024).

57 Pan, C. et al. Novel machine learning driven design strategy for high strength Zn Alloys optimization with multiple constraints. npj Computational Materials **11**, 169, (2025).

58 Rao, Z. et al. Machine learning–enabled high-entropy alloy discovery. Science **378**, 78-85, (2022).

59 Sasidhar, K. N. et al. Enhancing corrosion-resistant alloy design through natural language processing and deep learning. Science Advances **9**, eadg7992.

60 Chen, Z. Q., Shang, Y. H., Liu, X. D. & Yang, Y. Accelerated discovery of eutectic compositionally complex alloys by generative machine learning. npj Computational Materials **10**, 204, (2024).

61 Sohail, Y. et al. Machine-learning design of ductile FeNiCoAlTa alloys with high strength. Nature **643**, 119-124, (2025).
